# Effect of hypopressive and conventional abdominal exercises on postpartum diastasis recti: A randomized controlled trial

**DOI:** 10.1371/journal.pone.0314274

**Published:** 2024-12-12

**Authors:** Mercedes Soto-González, Iria Da Cuña-Carrera, Eva María Lantarón-Caeiro, Augusto Gil Pascoal

**Affiliations:** 1 Faculty of Physical Therapy, Department on Functional Biology and Health Science, University of Vigo, Pontevedra, Spain; 2 Galicia Sur Health Research Institute, Clinical Physiotherapy Group, Vigo, Spain; 3 Faculty of Human Kinetics, Interdisciplinary Centre for the Study of Human Performance, Biomechanics and Functional Morphology Laboratory, University of Lisbon, Lisbon, Portugal; Iran University of Medical Sciences, ISLAMIC REPUBLIC OF IRAN

## Abstract

**Background:**

Diastasis recti abdominis (DRA) is the separation of the rectus abdominis muscles along the linea alba, often occurring during pregnancy due to hormonal and mechanical changes. While DRA usually resolves post-childbirth, some women experience persistent issues. Exercise may help reduce the inter-rectus distance, though more research is needed.

**Objectives:**

Analyze the immediate and long-term effects of hypopressive and conventional abdominal exercises on inter-rectus distance (IRD) in postpartum women, focusing on parity differences.

**Methods:**

Twenty-eight women who met specific inclusion criteria were randomly assigned to either the hypopressive group (n = 14) or the conventional group (n = 14). The exercise programs for both groups lasted 6 weeks. Ultrasound measurements were taken of the IRD at two locations above the umbilicus, 2 cm (AB2) and 5 cm (AB5), before and after exercise programs.

**Results:**

The IRD decreased after both exercise programs compared to baseline measurements in AB2 (mean difference: 3.06 mm, 95% CI: 1.06 to 5.05) and in AB5 (mean difference: 2.88 mm, 95% CI: 1.59 to 4.17), confirming the long-term effect of exercise. No differences were found between the application of the conventional exercise program or the hypopressive exercises, indicating that both had a similar positive effect.

Regarding the immediate effect, after the performance of both programs, the IRD was greater during rest compared to abdominal contraction. This effect was more pronounced at AB5 location (mean difference: 1.00mm, 95% CI: 0.97 to 1.89). Conventional exercises result in a reduction during resting condition (mean difference: 4.52mm, 95% CI: 2.62 to 6.79), whereas with hypopressive exercises, the reduction occurs during muscle contraction (mean difference: 3.43mm, 95% CI: 1.21 to 5.65).

With regard to parity, multiparous women benefit most from the application of exercise programs.

**Conclusion:**

A reduction in IRD has been achieved with the implementation of both exercise programs in postpartum women, with multiparous women benefiting the most. Specifically, at 5 cm above umbilicus, conventional exercises result in a reduction during the rest condition, whereas with hypopressive exercises, the reduction occurs during muscle contraction.

**Trial registration:**

**Clinical trial registration**: NCT05439668.

## Introduction

Diastasis recti abdominis (DRA) is the midline separation of the two rectus abdominis muscles along the linea alba (LA) [1,2]. This condition is a physiological response during pregnancy, caused by hormonal changes in connective tissue elasticity and mechanical stresses on the abdominal wall due to fetal growth and displacement of abdominal organs [3,4]. In most cases, DRA resolves spontaneously after childbirth [5,6]. However, after childbirth, certain women do not undergo natural restoration, resulting in a pathological condition that can persist for several years [4,7].

The reviews were published to know the effect of exercise on diastasis recti [8–11] agree in the scarce existing literature and more research is needed. Nonetheless, some studies suggest that exercise programs may offer potential benefits in the treatment of DRA [11].

According to the literature, the abdominal crunch is the most extensively studied exercise for assessing the behaviour of the LA, and in the majority of cases, it leads to a decrease in the inter-rectus distance (IRD) compared to the resting stage [12–16].

Limited evidence exists regarding the impact of hypopressive exercises, with most research concentrating on their effects on the pelvic floor [17–21]. The impact of these exercises on the abdominal wall remains uncertain, but some studies [21–24] have observed an activation of the transverse abdominal muscle (TRAM) during hypopressive exercise [14,16,25]. This activation of the TRAM may also influence the IRD, leading to the hypothesis that hypopressive exercises could potentially affect this distance.

Therefore, the main purpose of our study was to analyze the effects of hypopressive exercise program compared with a conventional abdominal exercise program on postpartum IRD. Specifically, we investigated the immediate and long-term effects of the exercise programs. Secondly, we explored the differences between primiparous and multiparous women (parity).

## Methods

### Design

This multi-center parallel-group randomized controlled trial (RCT) was conducted in Vigo, Spain, from July to December 2022 to investigate the effects of two postpartum abdominal exercise programs on IRD. The trial followed the CONSORT guideline [26], was approved by the (Ethics Committee of Galicia South Health Research Institute) and registered at http://register.clinicaltrial.gov (NCT05439668). Participants provided written informed consent, and the study followed the Declaration of Helsinki guidelines.

### Participants

Among a total of 41 women who were contacted during prenatal consultations at public primary care centers in July 2022 (from July 1 to July 31, 2022), 28 participants were recruited for the study. The inclusion criteria were women who had undergone a vaginal delivery at least 8 weeks before the first measurement. This timeframe was chosen because the natural recovery of DRA is most pronounced between day 1 and 8 weeks postpartum [27]. Exclusion criteria included current pregnancy, abdominal hernia, and abdominal surgery (including C-section). The rationale for excluding these conditions is that surgery and hernias can cause structural changes that may affect abdominal muscle function [28]. Participants were allowed to withdraw voluntarily at any time, with the additional criterion of missing more than 20% of the exercise program sessions.

An a priori power analysis for sample size determination was conducted using G*Power software (version 3.1.9.2). We specified a power (1-β err prob) of 0.80, an α error of 0.05, two groups, four measurements, and an effect size (f) of 0.46. The correlation among repeated measures was set to 0.5, and the nonsphericity correction (ε) was fixed to 1. An effect size of 2 mm with a standard deviation (SD) of 7 mm was used for the experimental group, while for the control group, it was set at 2 mm with an SD of 6 mm. The between-group difference (95% CI) was estimated at 1 mm (−3 to 3). These values were derived from a previous study [29] that investigated the impact of a 12-week exercise program on postpartum inter-rectus distance (IRD) measured by ultrasound. The study compared differences in IRD between an intervention group and a control group of postpartum women, focusing on both between-group and within-group conditions (rest and curl-up) measured at 2 cm above the umbilicus.

Based on this analysis we estimated a sample size of 22 participants (11 per group). To account for a potential 30% loss to follow-up, a total of 28 participants (14 per group) were deemed necessary to ensure adequate power for the analysis. For the sample size calculation, the "as in SPSS" option was selected for effect size specifications in G*Power software.

All participants were equally randomized to both groups by a person who was not engaged in either the assessments or the intervention. Simple randomization was employed, using tables of random numbers, with an allocation ratio of 1:1. Hidden allocation methodology was implemented.

### Outcome measurement

The primary outcome measurement was the observed change in IRD, measured by ultrasonography. Regarding the immediate effects, the difference between the rest and contraction conditions is evaluated. Concerning long-term effects is the change from baseline (before exercise programs) to 6 weeks (after exercise programs).

### Instrumentation and procedures

Static ultrasound images were acquired following a valued protocol [30] using an ultrasound scanner (LOGIQ e; GE Healthcare, Waukesha, WI) with a 4 to 12-MHz, 39-mm linear transducer, set to a fixed frequency of 12 MHz in brightness mode (B-mode). Participants were positioned comfortably in a supine position, with knees flexed at a 90-degree angle and feet resting on the plinth while keeping their arms alongside the body.

All images were collected by a blinded physical therapist with specialized training in ultrasound imaging and previous experience in assessing IRD.

The images were captured with the ultrasound probe placed transversely along the midline of the abdomen, positioned at 2 cm and 5 cm above the umbilicus, while participants were in both a supine resting position and a crook lying position. To ensure consistent probe placement, the skin was marked with a water-soluble pen before each scan as shown in S1 Fig (The individual pictured in S1 File has provided written informed consent (as outlined in PLOS consent form) to publish their image alongside the manuscript).

In the crook lying position, participants were instructed to raise their heads and shoulders until both scapulae cleared the table. The images were taken at rest and during the isometric phase of the crook lying position, while participants maintained the position. During image acquisition, the probe was gently adjusted to optimize the visualization of both rectus abdominis muscles and LA. Images were collected immediately at the end of the exhalation following the recommendations of Teyhen et al. [31], and were exported in DICOM format for further offline processing.

The IRD was measured as the linear distance between the medial ends of both rectus abdominis muscles, i.e., the length of the straight line that connects the medial borders of the hyperechoic fascia surrounding both hypoechoic rectus abdominis muscles. To ensure consistency, the same investigator, who was blinded to the participant’s identification, measured the IRD offline using a customized code (Image Processing Toolbox, Matlab, Mathworks). The medial end points of both rectus abdominis muscles were identified using the same semi-automatic approach), employed in a previous study by Pascoal et al. [32]. An interpolated parabola-like curve was calculated using 8–12 digitized coordinates on the visible contour of each rectus abdominis. The parabola’s inflection point was used as a reference point for IRD measurement, guiding the examiner in identifying the medial end points of each muscle. The examiner made the final decision on selecting the reference points for IRD measurement, whether to follow the suggested parabola-like curve approach or not (S2 Fig).

It is important to note that the examiner retained the final decision on selecting the reference points on the muscle contour for IRD measurement, including the choice of following the recommended measurement point suggested by this parabola-like curve approach. After identifying the two reference points for measuring the IRD at the inner end of each rectus muscle, the respective coordinates in the ultrasound image were digitized. To calculate the IRD in millimeters, the number of pixels between these two coordinates was multiplied by the image pixel spacing value extracted from the DICOM metadata structure.

### Intervention

The intervention comprised a 6-week abdominal exercise program for each group, with a total of 12 sessions. One group undertook conventional abdominal exercises (e.g., curl-up), while the other engaged in hypopressive abdominal exercises. The classes, each lasting one hour, were conducted in person by two experienced physical therapists. All exercises were performed to relaxing music.

The hypopressive abdominal exercise program involves maintaining spine elongation, a neutral pelvis, knee flexion, and activation of scapular girdle muscles. Participants execute three normal breathing cycles with slow diaphragmatic inspiration, followed by complete air expiration, and subsequently hold their breath after rib-cage expansion (diaphragmatic suction) [33,34]. During the first two sessions, participants were instructed and learned breathing techniques and proper postures. In subsequent sessions, participants performed hypopressive exercises in different positions, including lying down, sitting, quadruped, and standing. Each position incorporated several exercise variations. Each exercise was performed in three repetitions, with a minimum apnea duration of 10 seconds. Participants rested for 30 seconds between sets.

The conventional abdominal exercise program included sit-ups, reverse sit-ups, bilateral leg raises, pelvic tilts, swimmer exercises, quadruped exercises, abdominal work exercises in standing position, and abdominal planks. Each exercise was performed during the expiratory phase and required the contraction of the pelvic floor muscles. Participants were instructed to perform between 1 to 3 sets of 10 repetitions per exercise, holding each contraction for 5 seconds followed by 10 seconds of relaxation. The number of sets and the difficult level were progressively adjusted over time.

Additional details regarding both exercise programs can be found in the S1 File.

### Data analysis

Statistical analyses were conducted using IBM SPSS for Windows version 28.0 (IBM Corp., Armonk, NY, USA), with a significance level set at p < 0.05 for all tests.

Data collected at each probe location (AB2 and AB5) were analyzed separately. A two-way ANOVA with repeated measures was employed to assess the effects of CONDITION (Rest vs. Abdominal Contraction) and MOMENT (Before vs. After) as within-subject factors, and GROUP (Conventional vs. Hypopressive) and PARITY (Primiparous vs. Multiparous) as between-subjects factors on IRD. When a significant main effect or interaction was found, pairwise t-tests with Bonferroni correction were used at each within-subject and between-subject factor. The assumption of sphericity was confirmed for all data, as evidenced by Mauchly’s Test of Sphericity (χ2(2) = 1.000), thus allowing us to use a two-way ANOVA with repeated measures without adjusting the degrees of freedom.

All effectiveness data were analyzed according to the intention-to-treat principle which means all participants were analyzed in the group to which they were randomized regardless of whether they received all or any of the treatment to which they were assigned.

## Results

### Participant flow and characteristics

**Fig 1:**
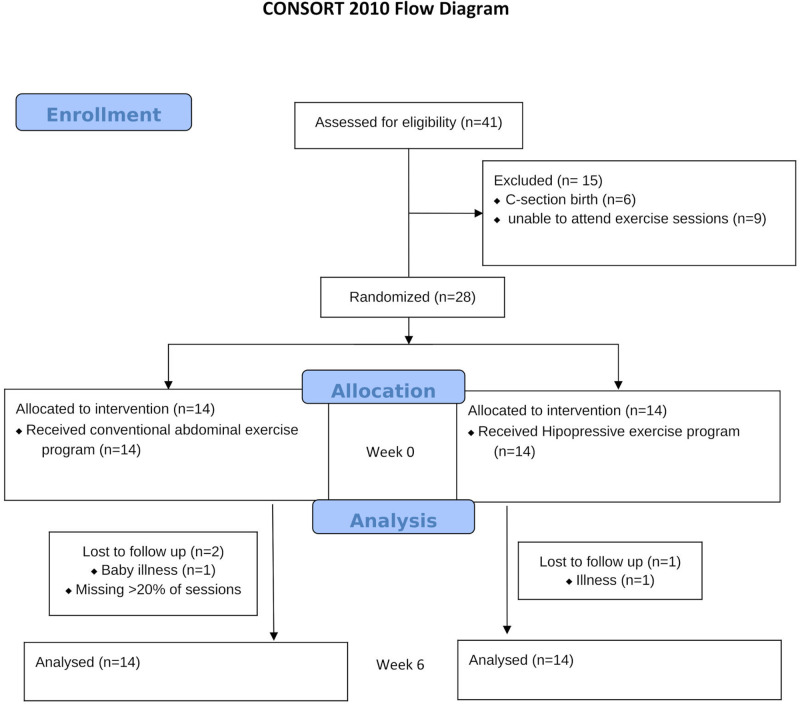
Flow chart of participants.

The study was conducted between July 2022 and December 2022. Twenty-eight eligible women who met the criteria agreed to participate in the study, with 14 women randomized to each group. Dropout rates and specific reasons for dropouts are detailed in Fig 1. Sample characteristics are displayed in Table 1, revealing no significant demographic or clinical differences between groups. The 95% confidence intervals for both Pregnancy Week Birth (-1.06 to 1.06 weeks) and Birth Weight (-0.49 to 0.12 kg) include negative values, suggesting that any observed differences could range from a decrease to a slight increase. Since the confidence intervals span zero and the means are close, it indicates that any potential differences are minimal and not statistically significant. This supports the conclusion that there is no meaningful difference in either birth weight or gestational age at birth between the two groups. No major or minor adverse events occurred during the exercise sessions and study period.

**Table 1:** Sample characteristics.

Sample Characteristics	TOTAL n = 28	GROUPS	
		CONVENTIONAL	HYPOPRESSIVE
		n = 14	n = 14
**AGE** **mean (SD), years**	34.21 (3.67)	33.35 (3.52)	35,07 (3.73)
**BMI** **mean(SD), Kg/m^2^**	25.83 (3.80)	26.61 (4.58)	25.04 (2.76)
**WEIGHT** **mean (SD), Kg**	69.82 (10.36)	71.66 (12.5)	67.96 (7.70)
**HEIGHT** **mean (SD), m**	1.65 (.05)	1.64 (.05)	1.65 (.05)
**Pregnancy Week Birth** **mean(SD), week**	39.64 (1.33)	39.64 (1.28)	39.64 (1.44)
**Postpartum Week** **mean (SD), week**	9.60 (2.37)	9.42 (2.82)	9.79 (1.93)
**Birth Weight** **mean (SD), Kg**	3,46 (0.40)	3.55 (.41)	3.37 (.38)
**Perineal Tear/Episiotomy** **n,(%)**	21 (75)	10 (71.4)	11 (78.6)
**Instrumental Delivery** **n, (%)**	11 (39.3)	5 (35.7)	6 (42.92)
**Breastfeeding** **n, (%)**	19 (67.9)	8 (57.1)	11 (78.6)
**Abdominal Exercise During Pregnancy** **n, (%)**	8 (28.6)	4 (28.6)	4 (28.6)
**Abdominal Exercise Before Pregnancy** **n, (%)**	13 (46.4)	7 (50)	6 (42.9)

BMI = Body Max Index;; SD = Standard Deviation.

#### Long-term effect of exercise on inter-rectus distance

This study aimed to analyze the changes in IRD after a 6-week abdominal exercise program, i.e. the long-term effect of exercise on IRD. Two groups of postpartum women, one following a Conventional exercise program and the other following a Hypopressive program, were studied to determine which exercise program is more effective in reducing IRD. The study also investigated whether the immediate reduction in IRD previously observed during abdominal contraction (e.g., curl-up) compared to the rest condition [3,35], i.e. the immediate effect of exercise on IRD, changed after the 6-week abdominal exercise program.

The long-term effect of exercise, in both hypopressive and conventional abdominal groups, was analyzed by comparing the inter-rectus distance (IRD) before and after the 6-week exercise programs (variable MOMENT).

A significant difference in IRD at 2 cm (AB2) and 5 cm (AB5) above the umbilicus was found between Moment 1 (week 0) and moment 2 (week 6) [F (1,24) = 10.02; p < .05; ηp2 = .29] and [F (1,24) = 121.29; p < .05; ηp2 = .47], respectively. Pairwise comparisons revealed that IRD significantly decreased over the 6-week exercise program, in both groups of participants at AB2 [Mean difference (SD) = 3.06 (5.13) mm; p < 0.04; CI95: 1.06 to 5.05] and AB5 [Mean difference (SD) = 2.88 (3.28) mm; p < 0.00; CI95: 1.59 to 4.17].

The effects size (ηp^2^ = 0.29 and 0.47) suggests a moderate impact of the exercise program on IRD.

When comparing the Conventional and Hypopressive abdominal exercise programs, no significant main effect of the GROUP variable on IRD was found at either AB2 or AB5. This indicates that both exercise programs had a similar impact on IRD changes observed between Week 0 and Week 6.

#### Immediate effect of exercise on inter-rectus distance

The immediate effect of exercise was analyzed by comparing IRD between REST and abdominal CONTRACTION (variable CONDITION).

A significant difference in IRD was found between REST and CONTRACTION conditions only in AB5 [F (1,24) = 5.23 p < .05; ηp2 = .18]. Pairwise comparisons revealed that IRD was significantly greater in the REST condition compared to the CONTRACTION condition [Mean difference (SD) = 1 (7,02) mm; p < 0.031; CI95: 0.97 to 1.89].

The immediate effect of exercise on IRD was more pronounced at the AB5 location, as indicated by a moderate effect size (ηp^2^ = .18).

#### Interactions between group, condition, and moment in IRD changes

At the AB5 location, a significant three-way interaction was found between variables GROUP, CONDITION, and MOMENT [F (1,24) = 6.01, p = 0.022, ηp^2^ = 0.20], indicating that the effects of Moment and Condition on IRD observed at AB5 vary depending on the Group. Overall, the moderate effect size (ηp^2^ = 0.20) suggests that 20.0% of the variance in IRD can be attributed to the combined effects of Moment, Condition, and Group.

As shown in Table 2, in the CONVENTIONAL exercise group, the IRD measured in the REST condition was significantly greater at Moment 1 (Week 0) compared to Moment 2 (Week 6) [Mean difference (SD) = 4.52 (5.82) mm; p < 0.001; CI95: 2.62 to 6.79]. This suggests a potentially beneficial long-term effect of the conventional exercise program on IRD additionally, in this group, the IRD was significantly greater in REST condition compared to CONTRACTION at Moment 1 [Mean difference (SD) = 1.94 (3.38) mm; p = 0.06; CI95: 0.6 to 3.26]. but no significant difference was found between REST and CONTRACTION at Moment 2.

**Table 2:** Mean (SD) of Inter-Rectus Distance (mm) measured 2 cm (AB2) and 5 cm (AB5) above the umbilicus, for both groups at rest (RT) and during an abdominal isometric contraction (AC), before (Week 0) and after (Week 6) the exercise program.

Probe Location	Group	Moment 1 (Week 0)		Moment 2 (Week 6)	
		RT Mean (SD)	AC Mean (SD)	RT Mean (SD)	AC Mean (SD)
**AB2**	**Conventional**	21.5 (8.99)	23.42 (9.20)	17.03 (7.94)	18,79 (8.31)
	**Hypopressive**	20.81 (8.46)	21.23 (8.68)	19.31 (7.46)	19.62 (7.83)
**AB5**	**Conventional**	20.97 (9.89)	**16.44**^#^ (8.15)	19.03* (10.95)	17.30 (9.95)
	**Hypopressive**	22.04 (9.31)	20.21 (7.67)	21.38 (10.32)	17.95*^#^ (9.42)

* The mean difference is significant (p < .05) between week 0 and week 6.

^#^ The mean difference is significant (p < .05) between RT and AC.

For the HYPOPRESSIVE Group, the IRD was significantly greater at Moment 1 compared to Moment 2 in the CONTRACTION condition [Mean difference (SD) = 3,43 (5.66) mm; p = 0.004; CI95: 1.21 to 5.65], indicating a decrease in IRD associated with the hypopressive exercises. Conversely, no significant difference was observed in IRD measured in the REST condition comparing the beginning (Moment 1) and the end (Moment 2) of the exercise program. Additionally, at Moment 2 in this group of participants, the IRD was significantly greater in the REST condition compared to CONTRACTION [Mean difference (SD) = 2.25 (5.55) mm; p = 0.004; CI95: 0.09 to 4.42], but there was no significant difference between REST and CONTRACTION at Moment 1.

#### Interactions of parity, group, moment, and condition on IRD changes

Parity significantly influenced the results on both AB2 and AB5 locations.

At the AB2 location, a significant three-way interaction was found between variables GROUP, PARITY, and CONDITION on IRD [F (1,24) = 8.76, p < 0.05, ηp^2^ = 0.267], indicating that the immediate effect of exercise (CONDITION)) vary depending on Group and Parity. The partial eta squared (ηp^2^ = 0.267) indicates a moderate to large effect size, suggesting that 26.7% of the variance in IRD can be attributed to the interaction between Group, Parity, and Condition.

Specifically, multiparous women in the conventional exercise group exhibited reduced IRD during abdominal contraction [Mean difference (SD) = 4.63 (7.4) mm; p < .05; CI95: 1.74 to 7.52), while primiparous women did not show this change.

**Fig 2:**
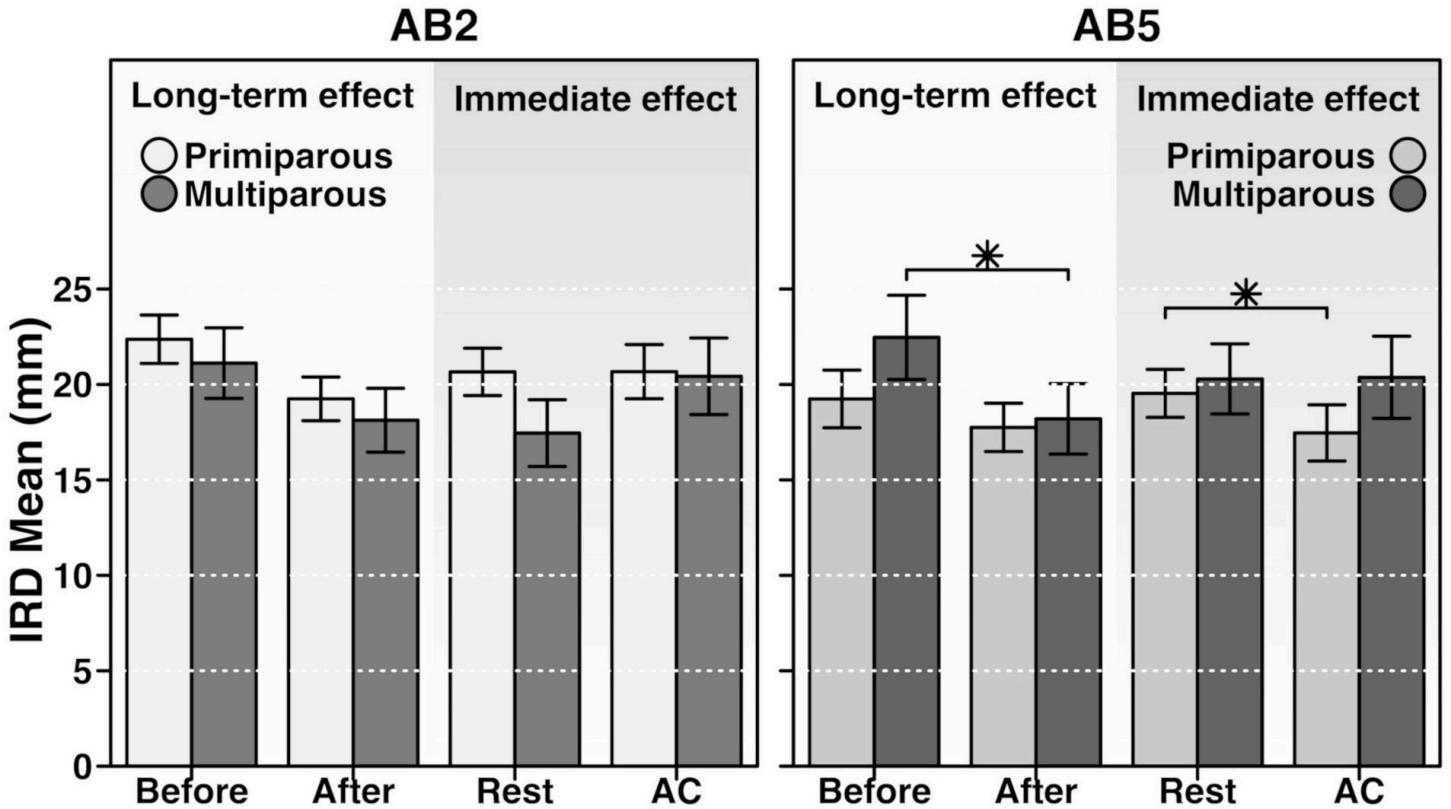
The immediate and long-term effects of exercise on postpartum inter-rectus distance (IRD) in primiparous versus multiparous women, measured 2 cm (AB2) and 5 cm (AB5) above the umbilicus. For the immediate effect, the Interrectus distance (IRD)in the Rest condition was compared with IRD in Abdominal Contraction (AC). For the long-term effect, IRD before the intervention was compared with the IRD after the intervention. An asterisk indicates cases where the comparison by ANOVA test returns a p-value < 0.05. AB2 and AB5 means 2 and 5 cm above umbilicus respectively.

At the AB5 location, a significant interaction was found between variables PARITY and MOMENT [F(1,24) = 4,94, p <0.05, ηp^2^ = 0.17] and between PARITY and CONDITION [F(1,24) = 6,17, p <0.05, ηp^2^ = 0.20] (Fig 2).

Pairwise comparisons revealed that for multiparous, the IRD at Moment 1 was significantly greater than at Moment 2 [Mean difference (SD) = 4.27 (5.45) mm; p < .00; CI95: 2.14 to 6.39]. The moderate effect size (ηp^2^ = 0.17) indicates that 17% of the variance in IRD could be attributed to the interaction between Moment and PARITY for primiparous. No significant interaction was found between MOMENT and PARITY for primiparous.

Pairwise comparisons revealed that for primiparous individuals, IRD was significantly greater at REST compared to CONTRACTION [Mean difference (SD) = 2.07 (2.59) mm; p = 0.00; CI95: 1.06 to 3.09]. The partial eta squared (ηp^2^ = 0.20) indicates a moderate effect size, suggesting that 20% of the variance in IRD is attributed to the interaction between condition and parity.

## Discussion

The primary objective of this study was to assess both the immediate and long-term effects of exercise on postpartum supraumbilical inter-rectus distance (IRD). Immediate effects were evaluated by comparing IRD measurements taken at rest and during an abdominal isometric contraction. Long-term effects were analyzed by comparing IRD measurements before and after a 6-week intervention involving two distinct abdominal strengthening programs: a Conventional program and a Hypopressive program. Additionally, the study aimed to determine the effectiveness of these programs in reducing IRD over 6 weeks and to examine how these effects varied based on the participant’s parity status, whether they were primiparous or multiparous.

### Long-term effect of exercise on inter-rectus distance

Concerning the long-term effect of abdominal exercises on the IRD, our results showed a reduction in IRD for both groups. This outcome aligns with the potential therapeutic benefits of exercise programs for treating DRA as documented in existing literature [8,10,11].

Furthermore, our results are consistent with those from several RCTs that have investigated the impact of conventional abdominal strengthening programs on IRD. Despite variations in program duration, frequency, and exercise techniques, these studies consistently demonstrate positive outcomes in reducing IRD with abdominal exercises [36–39], which is in line with our findings. Conversely, in certain RCTs [10,29,40], no reduction in IRD was reported in association with exercise. These differences in outcomes could be attributed to variations in exercise frequency across the studies. Specifically, those showing no reduction in IRD may have had exercise sessions supervised only once per week [40,41], or lacked supervision session [29]. In contrast, the majority of studies that achieved a reduction in IRD typically included three supervised sessions per week [36–39].

Another noteworthy finding from our study was that women participating in a conventional abdominal exercise program, in contrast to those engaged in the hypopressive program, effectively reduced their IRD at rest. This outcome suggests a potentially beneficial impact of the conventional program on baseline abdominal muscle tone favoring IRD reduction [29]. Further investigation into the effects of exercise programs concerning women’s parity, a significant reduction in IRD was observed exclusively at the AB5 location when comparing before-after intervention, particularly among multiparous women. This finding suggests that multiparous women may experience greater benefits from exercise programs, possibly attributed to commencing with tissue overstretching, resulting in more pronounced improvements both pre- and post-intervention compared to primiparous women.

In this sense, Werner et al. [42] demonstrated that parity increases the risk and prevalence of DRA due to repeated stretching and hyper-pressure of the abdominal wall. This finding suggests the presence of irreversible or unrecoverable stretching of the connective tissue, leading to plastic tissue deformation along the direction of the collagen fibers [43,44]. Further, Mota et al. [45] identified a greater separation of the IRD at AB2 compared to AB5 during pregnancy. This difference could potentially result in increased traction forces at that abdomen level, resulting in increased irreversible deformation. This observation may offer insights into the disparities observed in our study.

Several studies [12,14,16,46] have shown that contracting the abdominal muscle leads to a decrease in IRD compared to measurements taken at rest and during an abdominal muscle contraction while lying in a supine position. This immediate effect of the exercise is particularly evident during the semi-curl-up exercise, also known as the abdominal crunch, performed in a crook lying position.

In our study, we observed a decrease in IRD measured 5cm above umbilicus (AB5) when the abdominal muscles were contracted. This observation aligns with the results from other studies which have consistently reported a reduction in IRD at the end position of the semi-curl-up exercise [12,14,46]. Regarding parity, significant differences were only observed in the AB5 location among primiparous women, who showed the greatest reduction in IRD during abdominal muscle contraction. This observation may also be linked to the tissue stretching experienced by multiparous women [42].

At the AB2 location, multiparous women in the conventional exercise group experienced an increase in IRD during contraction. This finding contradicts results from other studies analyzing the immediate effect of abdominal contraction on IRD [16,16,46].

This discrepancy may arise from the initial condition of the tissues in multiparous women, which often starts with a lower LA stiffness [47]. This initial distension might render them less competent and less capable of effectively reducing their IRD to generate tension in response to abdominal hyper-pressure, as observed in other studies [13]. This observation suggests that multiparous women may face greater challenges in responding to abdominal muscle contractions effectively. However, it may also explain why these women derive greater benefits from exercise programs, as previously indicated.

Limited research has been conducted to assess the immediate effect and long-term effects of hypopressive abdominal exercises on Diastasis Recti Abdominis (DRA). Ramírez et al. [48] were among the first researchers to explore the long-term impact of a hypopressive abdominal exercise program on reducing IRD in postpartum women. Their study involved women who were 8 weeks postpartum with an initial IRD measurement exceeding 2 cm. Their results, alongside those of a prior case study [49], were consistent with our findings, showing a reduction in IRD following an exercise program compared to baseline measurements. It’s worth noting that in our study, the most significant effect was observed during the contraction condition when comparing the IRD before and after. This suggests a potential beneficial effect of the hypopressive exercises on the anticipatory activation of the deep abdominal muscles.

In 2021, Da Cuña-Carrera et al. [50] observed no statistically significant differences when comparing rest to performing hypopressive abdominal exercises. This suggests that such exercise may not have an immediate effect on DRA. However, Arranz-Martínez et al. [46] observed an increase in the IRD above the umbilicus and a decrease below the umbilicus. This seems to indicate that the effect of hypopressive exercises on IRD may vary depending on the location of the DRA.

Some studies [46,48] suggest that the effects of hypopressive exercises on the postpartum abdominal female musculature extend beyond their impact on IRD. These studies propose considering parameters such as tension on the linea alba (LA) for inclusion in future research. Lee & Hodges [13] introduced the concept of the "distortion index" to describe the behavior of the LA during the contraction of the abdominal muscles in response to exercises. The distortion index measures the average deviation of the LA’s path from the shortest route between its attachments. This deviation reflects the tension or stiffness of the LA, with greater distortion occurring when the LA is less tense or stiff. The calculation of the distortion index involves dividing the area enclosed by the LA’s path and the IRD by the IRD itself. Specifically, when the LA distorts anteriorly, the anterior boundary of the fascia is traced, and when it distorts posteriorly, the posterior boundary is traced. Tissue stiffness is then assessed within the area delineated by the superficial and deep borders of the LA. Therefore, even if there is no reduction in IRD during the execution of an abdominal exercise, it can still be beneficial if the distortion index is low. A low distortion index suggests that the LA is maintained under tension, indicating effective engagement of the abdominal muscles despite the absence of changes in IRD. Based on this theoretical framework, although hypopressive abdominal exercises may lead to an increase in IRD due to the activation of the TRAM, this could indicate favorable conditions for the recovery of the LA. The reduction in distortion index and the alignment of the LA with its attachments suggests that despite the IRD increase, the LA is under tension, potentially promoting its recovery.

Despite valuable insights provided by our study on the effects of different abdominal muscle exercises on IRD in postpartum women with and without DRA, it’s important to recognize specific limitations in interpreting the findings. One such limitation is the inclusion of participants regardless of their initial IRD values, which may have affected the observed outcomes. It is known that the behavior of the LA can differ between women with and without DRA [47]. Another limitation is the lack of a control group in our comparison of exercise programs, which hinders us from determining whether the observed changes in IRD are solely attributed to the exercises or partially influenced by the natural resolution of DRA after childbirth [4]. The effect of exercise on IRD should be analyzed by comparing intervention effects with untreated controls due to the natural resolution of DRA in the postpartum period [7]. Seven randomized trials have investigated this comparison. Two of these studies [40,41] found no significant impact from exercise interventions involving transversus abdominis activation or pelvic floor muscle training combined with abdominal exercises. Measurement methods, such as palpation, complicated direct comparison with the current results. Conversely, five other trials [51–55] reported beneficial effects on IRD compared to untreated controls, with participant profiles similar to those in this study, particularly regarding parity and the timing of postpartum training onset. However, these studies indicate that targeted exercise programs can effectively reduce IRD postpartum. However, these studies had limitations, including small sample sizes (e.g., n = 10 in Tuttle et al., 2018) and differing cut-off values for DRA [53]. Notably, only one study [51], which is a pilot trial, used ultrasound for IRD measurement.

Furthermore, the measurements in our study were taken in a supine position, a common approach in research and consistent with a recent scoping review [56]. However, for a more comprehensive understanding of IRD during dynamic abdominal muscle exercises and its functional implications, future studies should consider measuring IRD in upright positions such as sitting and standing. Previous research has shown wider IRD values in the standing position compared to the supine position [57].

Future research should encompass an exploration of both the immediate and long-term effects of a wide range of abdominal strength exercises, including hypopressive exercises, on Linea Alba (LA) morphology and mechanical behavior. This could be achieved by utilizing the distortion index parameter, as proposed by Lee & Hodges [13] but also by employing ultrasound elastography techniques to assess the stiffness of the LA and/or deeper abdominal muscles. Furthermore, it is essential to investigate the correlation between IRD reduction in postpartum women and the mechanical behavior and adaptation to exercise of the connective tissue that composes the LA.

The study results, coupled with proposed advancements in research methodologies such as distortion index analysis and ultrasound elastography, hold promise for creating personalized rehabilitation approaches for postpartum women with diastasis recti abdominis. These methodologies empower clinicians to craft more impactful exercise programs and interventions, leading to better clinical results and an improved quality of life for postpartum women.

## Conclusion

A reduction in IRD has been achieved with the implementation of both 6-week exercise programs (conventional and hypopressive) in postpartum women, with multiparous women benefiting the most. Specifically, at 5 cm above the umbilicus, conventional exercises result in a reduction during the rest condition, whereas with hypopressive exercises, the reduction occurs during muscle contraction.

## Supporting information

S1 Checklist (DOCX)

S1 Fig Skin marks is the S1 Fig title. (JPG)

S2 Fig Inter-rectus-distance measurement is the S2 Fig title. (JPG)

S1 File Exercise programs is the S1 File title. (DOCX)

S1 Data (XLSX)

S2 Data (XLSX)

S1 Protocol (PDF)

S2 Protocol (PDF)
