# Supplementary figures and images for: Effect of hypopressive and conventional abdominal exercises on postpartum diastasis recti: A randomized controlled trial
Source: PLoS One. 2024 Dec 12;19(12):e0314274. doi: 10.1371/journal.pone.0314274 (PMC11637234; doi:10.1371/journal.pone.0314274)

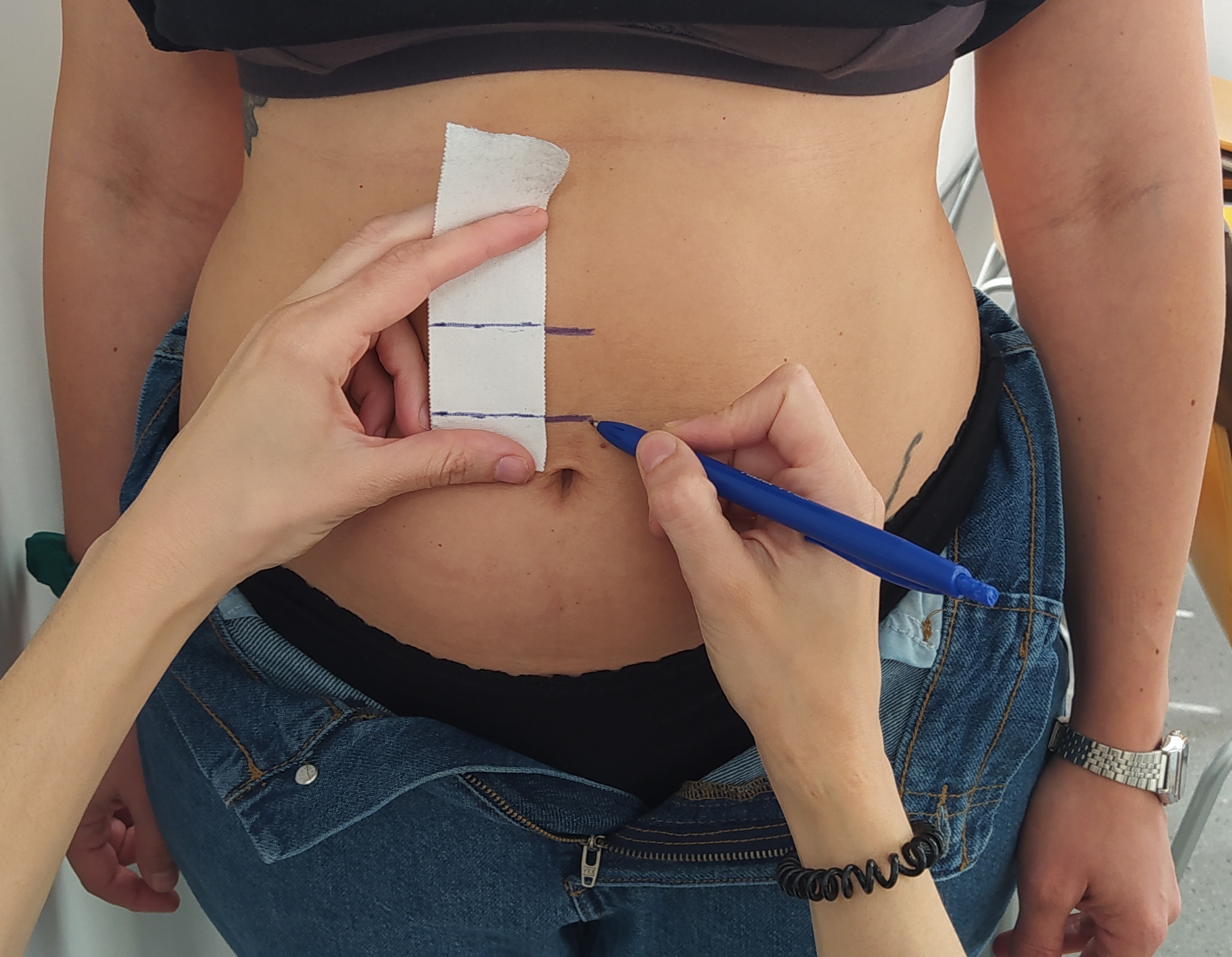

Supplement: S1 Fig — (JPG) [file pone.0314274.s002.jpg]

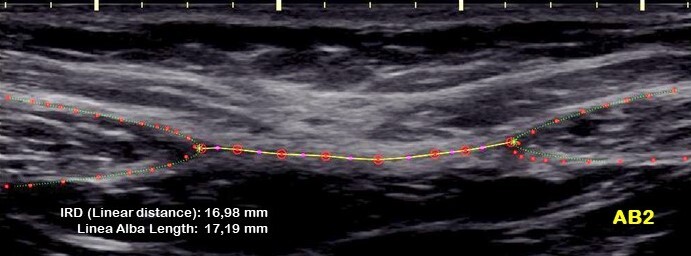

Supplement: S2 Fig — (JPG) [file pone.0314274.s003.jpg]
