# Supplementary material for: Effect of hypopressive and conventional abdominal exercises on postpartum diastasis recti: A randomized controlled trial
Source: PLoS One. 2024 Dec 12;19(12):e0314274. doi: 10.1371/journal.pone.0314274 (PMC11637234; doi:10.1371/journal.pone.0314274)
Supplement: S1 File — (DOCX) [file pone.0314274.s004.docx]

# CONVENTIONAL ABDOMINAL STRENGTHENING EXERCISES

All exercises are performed during the expiratory phase and involve the contraction of the pelvic floor muscles. The exercises are described as follows.

| Exercise | Procedure |
| --- | --- |
| Sit-Up | The woman assumes the supine position with her hips and knees flexed while her feet are supported. She is then instructed to raise her trunk until she can touch her knees with her hands. This exercise was performed with the following variations: raising both knees and trunk simultaneously or providing support to the lower limbs using a fitball. |
|  | 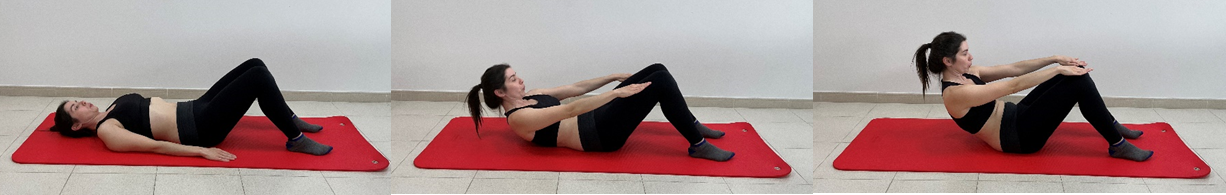 |
|  | 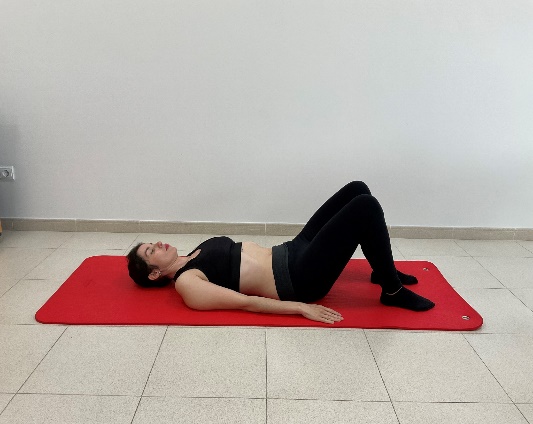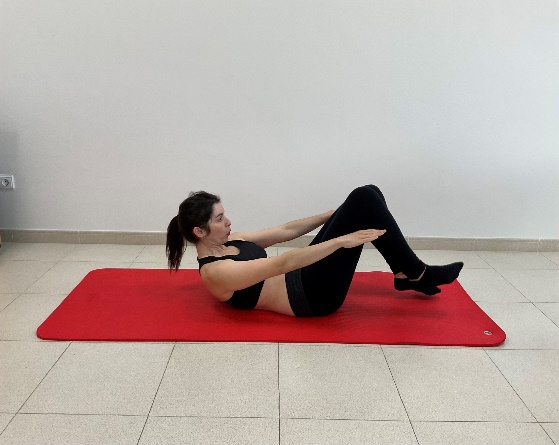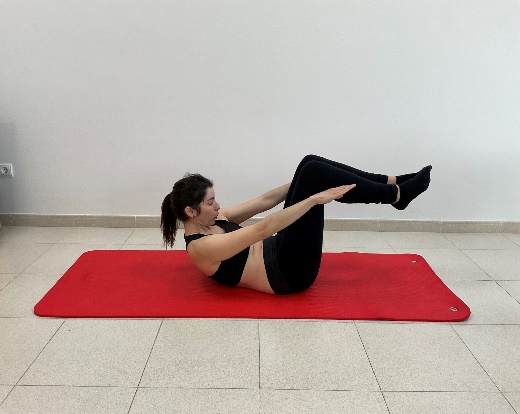 |
|  | 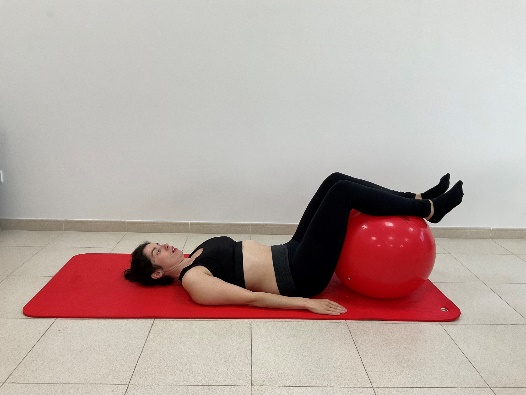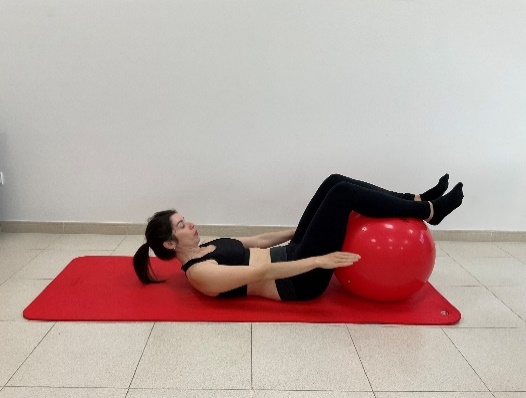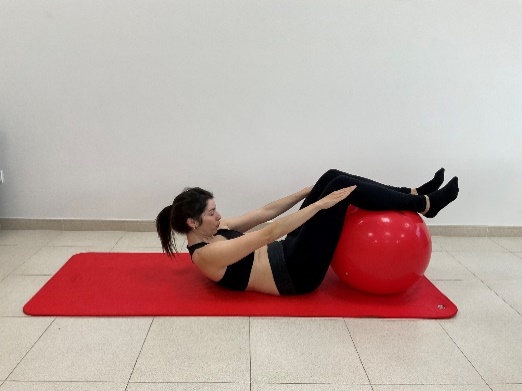 |

| Reverse Sit-Up | The exercise starts with the woman seated, her hips and knees flexed, and feet supported. From this position, she performs a trunk extension, which involves an eccentric contraction of the abdominal muscles.  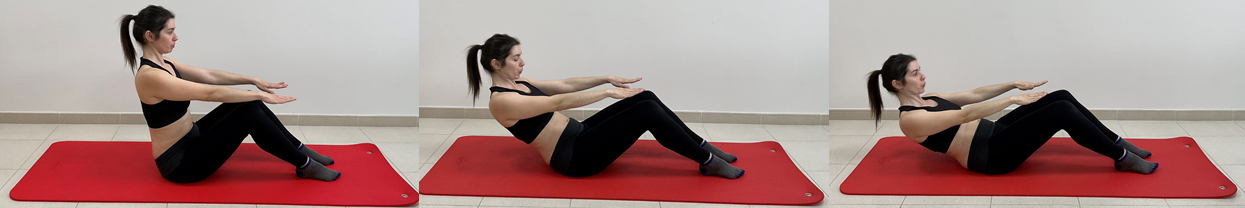 |
| --- | --- |
| Bilateral Leg Raise Exercise | The exercise begins with the woman in a supine position, with her hips and knees flexed. She is then instructed to raise her legs towards her trunk. This exercise has also been performed with her legs extended.  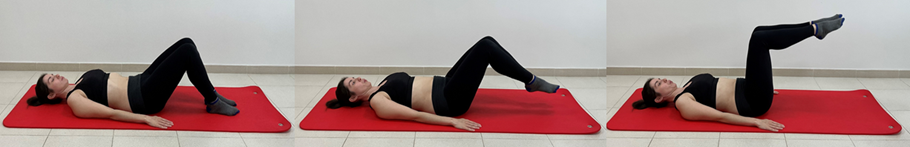  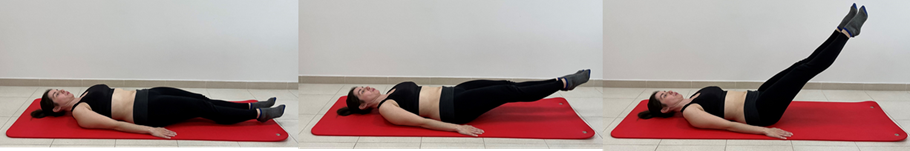 |

| Pelvic Tilts | Retroversion and anteversion pelvic tilts are performed in both supine and standing positions.  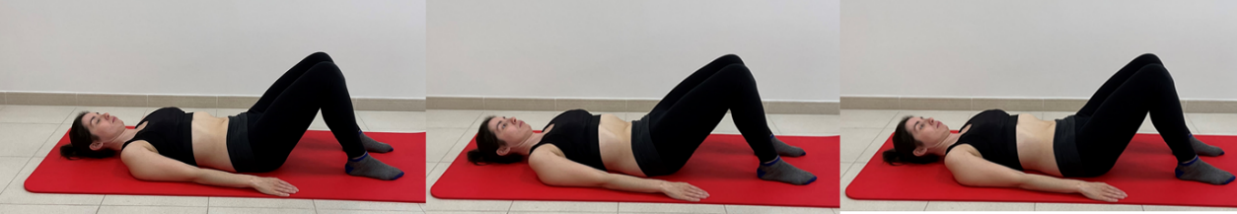 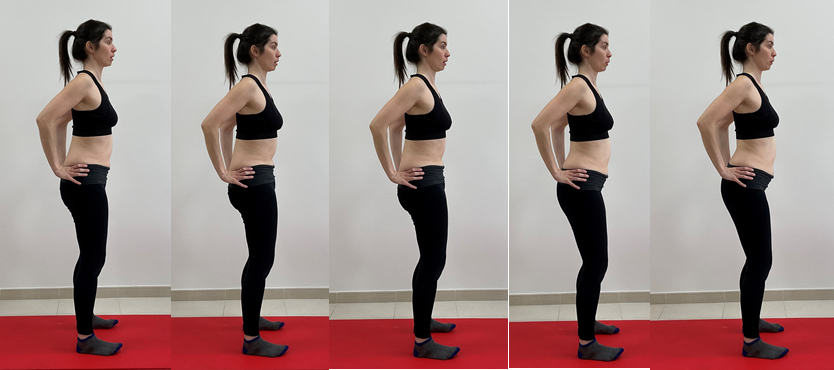 |
| --- | --- |

| Swimmer Exercise | The woman is positioned in a prone position and instructed to extend one lower limb and the opposite upper limb. This exercise is also performed by lifting both arms simultaneously or both legs simultaneously  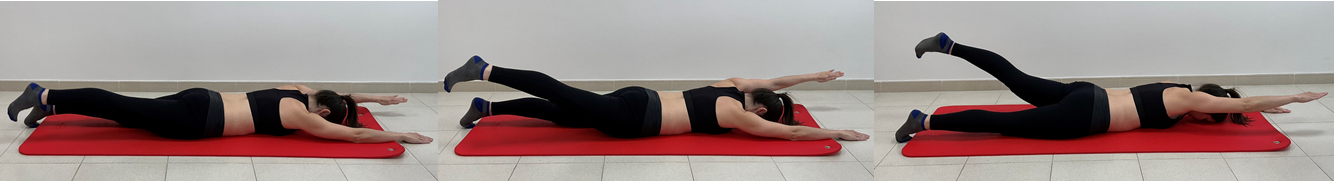  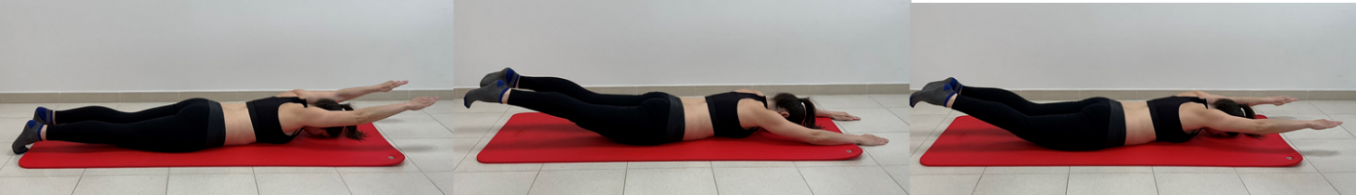 |
| --- | --- |
| Exercise In Quadruped Position | The exercise begins in a quadruped position, i.e., with both knees and hands on the ground. Next, the woman is instructed to lift one upper limb (arm flexion) while simultaneously lifting the contralateral lower limb (hip extension). 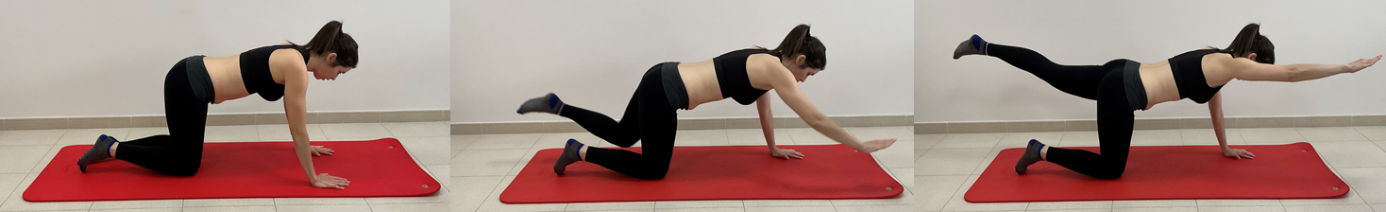 |

| Abdominal Work Exercise in Standing Position | The hip elevation is performed together with trunk flexion. The woman should also touch her foot with her same-side hand.  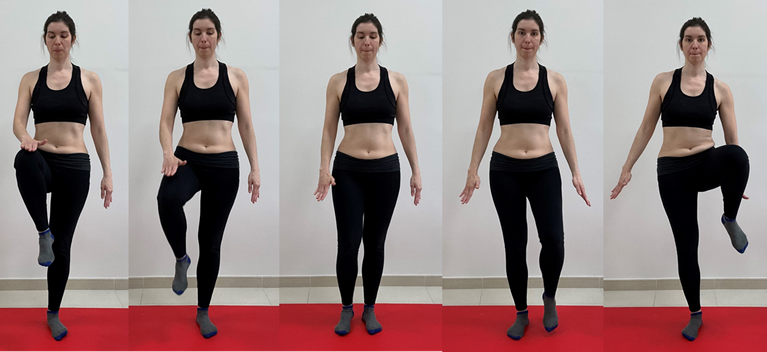 | |
| --- | --- | --- |
| Abdominal Plank Exercise | The performance of an abdominal plank (on elbows and toes) is sought, increasing its duration as the sessions progress (up to 20 seconds). | 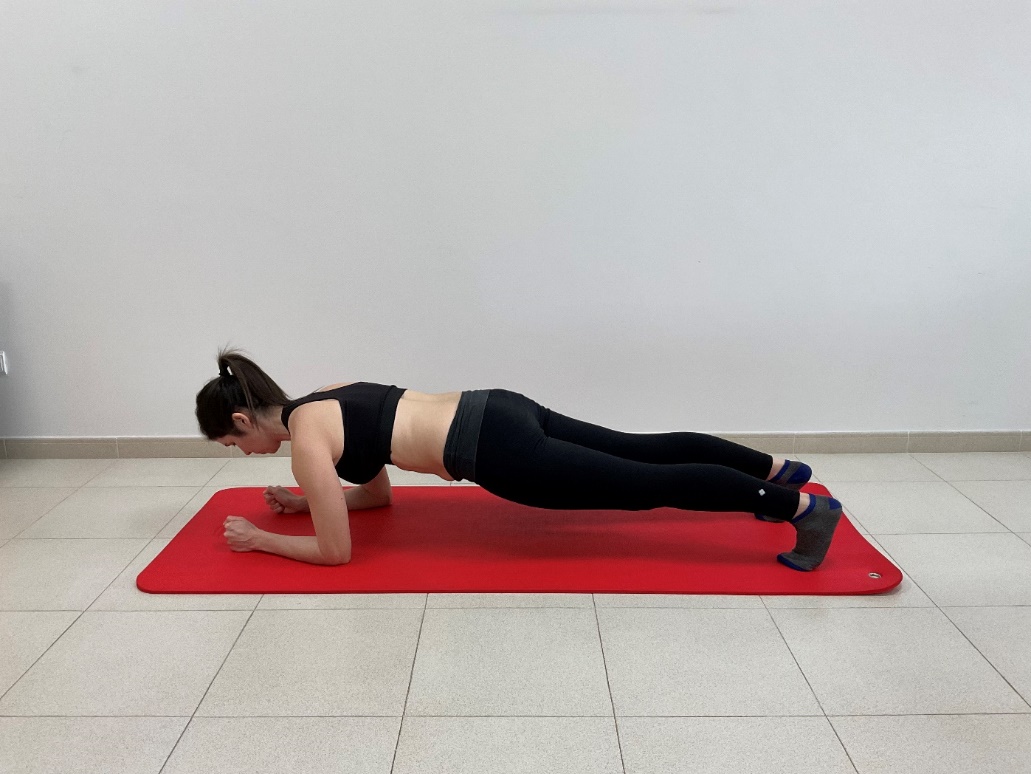 |

# ABDOMINAL HYPOPRESSIVES EXERCISES

Hypopressive exercises involve spine elongation, neutral pelvic, knee flexion, and scapular girdle muscle. Patients do three normal breathing cycles with slow diaphragmatic inspiration and a total air expiration followed by apnea-holding after rib-cage expansion (diaphragmatic suction). The first two sessions consisted of learning breathing techniques and postures and in the following sessions, hypopressive exercises were done in different positions.

The hypopressive exercises were performed lying down, sitting down, in the quadruped, and standing position, having several variants in each position. The following is a detailed description of how they were performed:

| Exercise | Procedure |  |
| --- | --- | --- |
| Static hypopressive exercise in the supine position | The woman lies on her back on a mat, having her hips and knees flexed, heels supported and the pelvis in a neutral position. The upper limbs are positioned with a slight shoulder abduction, the elbows are flexed, and the wrists are extended, so that the hands are held at hip level. Axial self-elongation, activation of the scapular stabilizing musculature, and decoaptation of the glenohumeral joint are sought. Once the position is maintained, the hypopressive technique of diaphragmatic suction is performed as described above. | 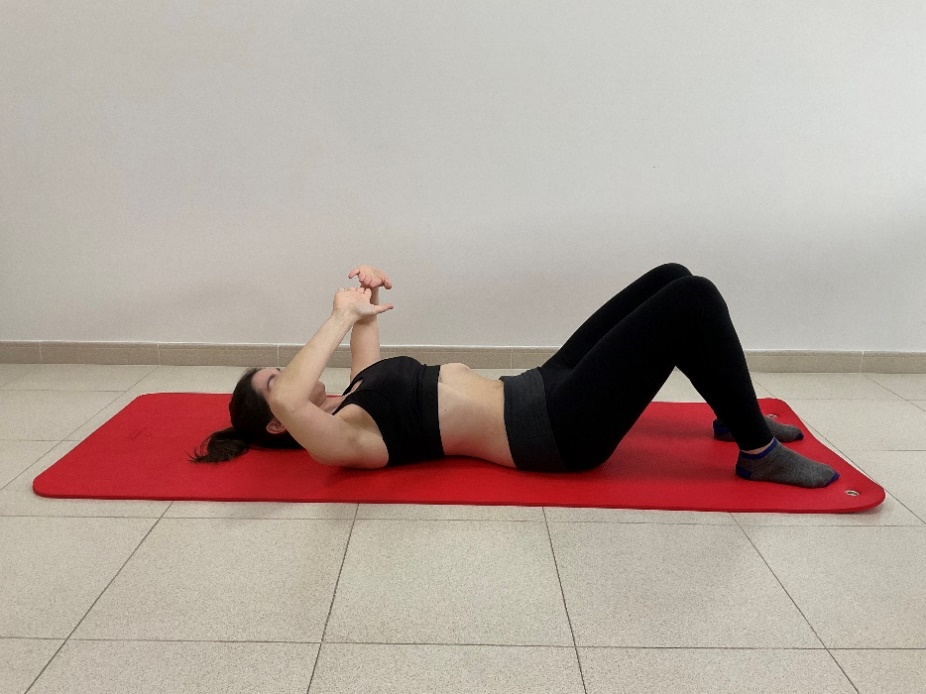 |
| Dynamic hypopressive exercise in a supine position | The woman is positioned in the same way as in the static exercise in a supine position, but once she performs the hypopressive technique, being in expiratory apnea, the elevation of a lower limb is sought. The exercise is then repeated with the elevation of the contralateral lower limb. Also, pelvic elevation is sought in an exercise variant when she is in respiratory apnea. | 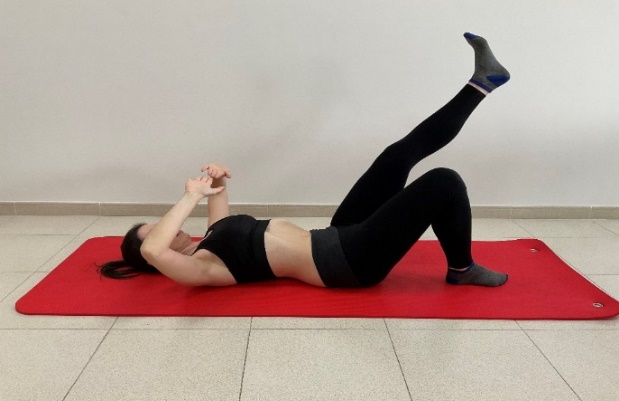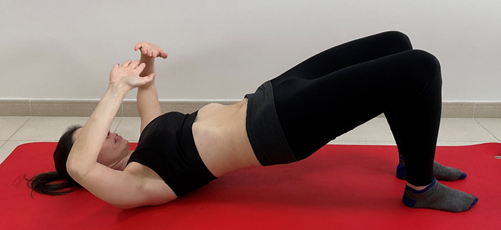 |
| Seated static hypopressive exercise | The woman is seated cross-legged. The upper limbs are positioned with slight shoulder abduction, the elbows are flexed, and the wrists are extended, so that the hands are kept at hip level. In this position, axial self-elongation, activation of the scapular musculature, and decoaptation of the glenohumeral joint are sought. Once the posture is maintained, the hypopressive technique is performed with diaphragmatic suction | 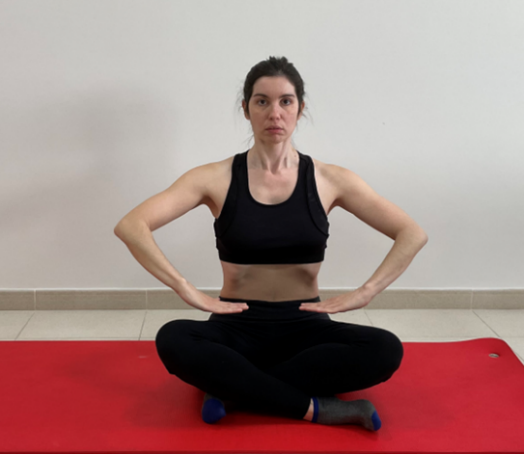 |
| Dynamic seated hypopressive exercise | The woman is positioned cross-legged, like the static seated hypopressive exercise. The hypopressive technique is then performed in the same manner as the previous exercise, and during the expiratory apnea, an upper limb is elevated. This exercise is then repeated with the other upper limb elevated. | 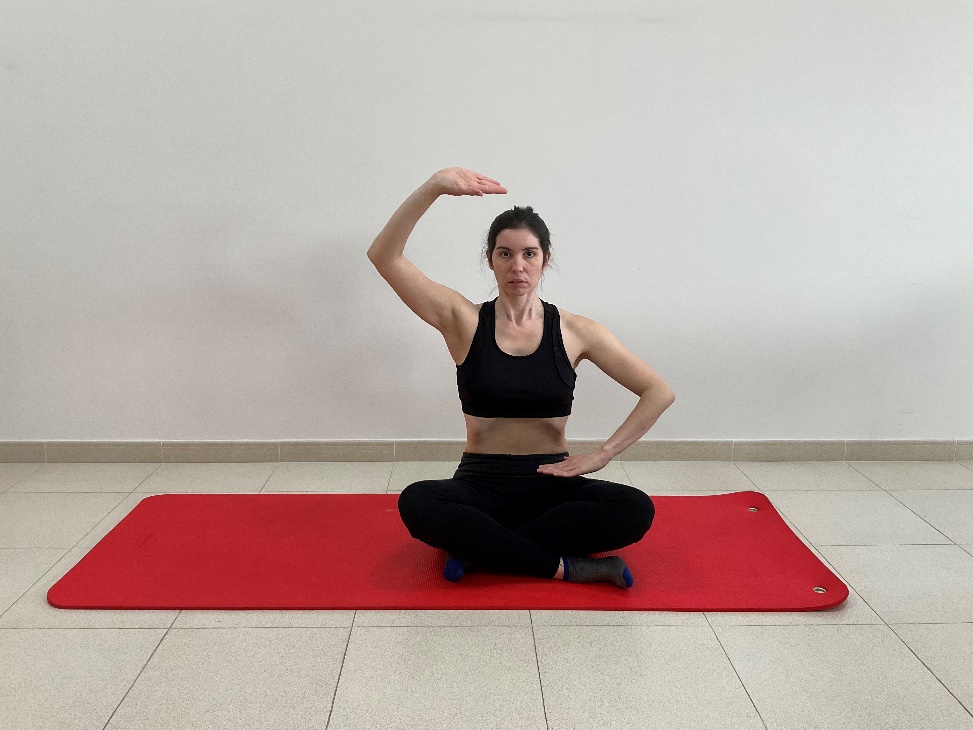 |

| Static hypopressive exercise in quadruped | The woman is positioned in a quadruped position with support on her hands, knees, and feet. The fingers of the hands are placed facing each other and the elbows are semi-flexed. The pelvis is kept in a neutral position and axial self-elongation, activation of the scapular stabilizing musculature, and decoaptation of the glenohumeral joint are sought. In this position, the hypopressive technique of diaphragmatic aspiration is performed. | 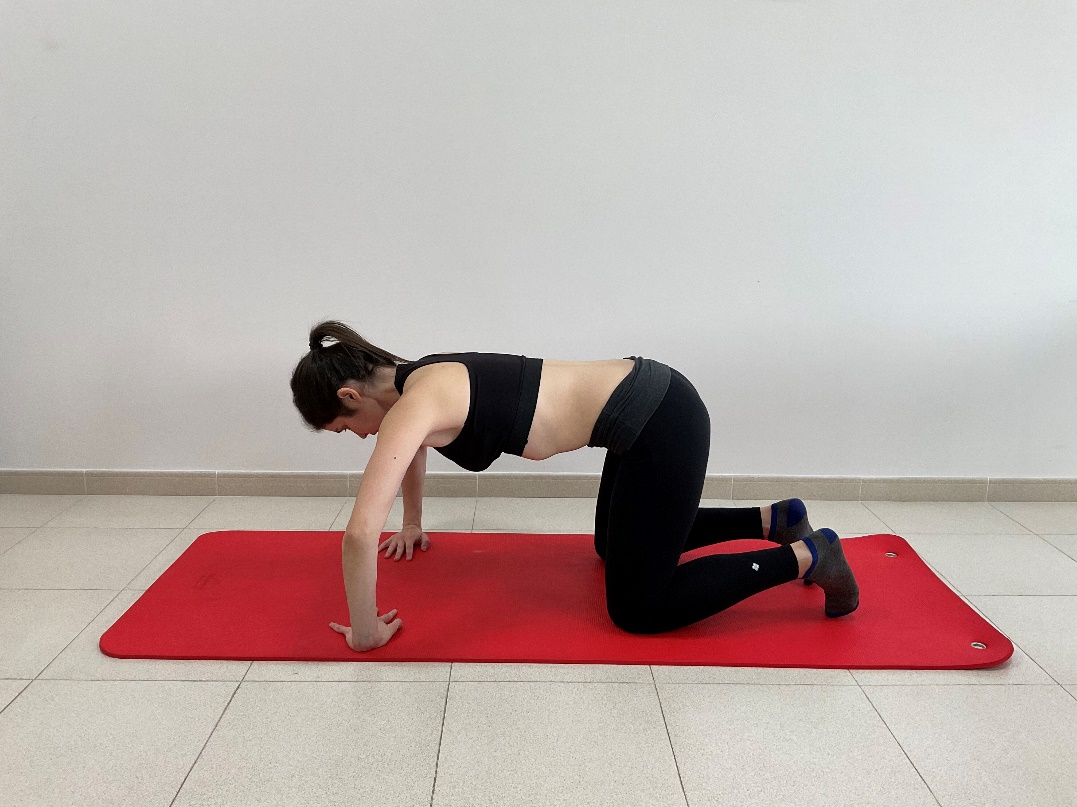 |
| --- | --- | --- |
| Dynamic hypopressive exercise in quadruped | The woman performs the static hypopressive exercise in quadruped, and while she is in expiratory apnea, she elevates one of the lower limbs. This exercise is repeated with the contralateral lower limb. | 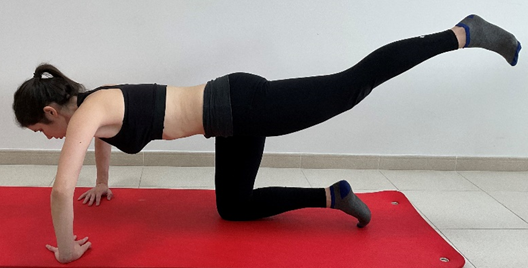 |

| Static hypopressive exercise in a standing position | The woman is in a standing position, with a neutral pelvis, lower limbs slightly apart (at the height of the hip joint), and a slight knee flexion. Self-elongation, activation of the scapular stabilizing musculature, and decoaptation of the glenohumeral joint are sought. The arms are placed in internal rotation, with a slight flexion of the elbows and with the hands facing each other with wrist extension. A forward movement of the body axis is sought. The hypopressive technique is performed in this position. | 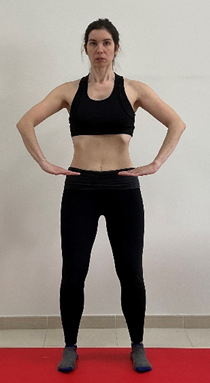 |
| --- | --- | --- |
| Dynamic hypopressive exercise in standing position | The static hypopressive exercise is performed in a standing position and, during the diaphragmatic suction phase in respiratory apnea, the upper limbs are lifted to the front up to shoulder height. | 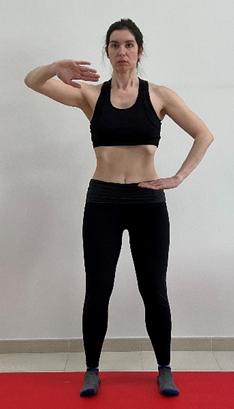 |
