# Supplementary material for: Effect of hypopressive and conventional abdominal exercises on postpartum diastasis recti: A randomized controlled trial
Source: PLoS One. 2024 Dec 12;19(12):e0314274. doi: 10.1371/journal.pone.0314274 (PMC11637234; doi:10.1371/journal.pone.0314274)
Supplement: S1 Protocol — (PDF) [file pone.0314274.s007.pdf]

PROTOCOLO DE INVESTIGACIÓN

COMPARACIÓN DE DOS PROGRAMAS DE EJERCICIOS ABDOMINALES SOBRE LA DISTANCIA INTERRECTOS EN MUJERES EN EL POSTPARTO

Iria Da Cuña Carrera

Enero de 2022

- **Título del proyecto**: COMPARACIÓN DE DOS PROGRAMAS DE EJERCICIOS ABDOMINALES SOBRE LA DISTANCIA INTERRECTOS EN MUJERES EN EL POSTPARTO

- **Código protocolo**

- **Versión de protocolo con fecha**

- **Promotor/a:** Iria Da Cuña Carrera

- **Investigador/a principal:** Iria Da Cuña Carrera. Profesora ayudante doctora en la Facultad de Fisioterapia en la Universidad de Vigo.

Datos de contacto:

Iria Da Cuña Carrera.

Correo electrónico: iriadc@uvigo.es.

Teléfono: 986801758 / 652131637.

Facultad de Fisioterapia. Campus a Xunqueira s/n CP36005 Pontevedrea

- **Investigadores/as colaboradores**
  - Eva M. Lantarón Caeiro (Universidade de Vigo)
  - Mercedes Soto González (Universidade de Vigo)
  - Augusto Gil Pascoal (Universidade de Lisboa)
  - Maria Ameijeiras Canosa (Centro de salud de Sárdoma, Matamá y Bembrive)
  - Ana Isabel Costalago Herrera (CS Navia)
  - Josefa Pereira Perez (CS Coia)
  - Carmen López Videla (C.S Teis)
  - Sara Arias Freire (CS Coia)

Índice

1. **Antecedentes y justificación del proyecto .... Pág. 4-5**
2. **Hipótesis del estudio .... Pág. 5**
3. **Objetivos .... Pág. 5**
4. **Tipo de estudio .... Pág. 5**
5. **Material y métodos .... Pág. 5-9**
   5.1. Ámbito de estudio
   5.2. Definición de los sujetos a estudio
   5.3 Captación y reclutamiento de los participantes
   5.4 Procedimiento de asignación/aleatorización
   5.5 Justificación del tamaño muestral
   5.6 Variables principal y secundarias
   5.7 Descripción de la intervención
   5.8 Cronograma y fecha prevista de finalización. Distribución de las tareas entre los miembros del equipo investigador
   5.9 Plan de análisis estadístico
6. **Aspectos ético-legales .... Pág. 10-11**
   6.1 Modelos de consentimiento informado.
   6.2 Confidencialidad de la información recogida en el contexto del estudio.
   6.3 Póliza de seguro o justificación de su ausencia para los estudios experimentales
   6.4 Compromiso de publicación de los resultados
7. **Memoria económica y fuente de financiación .... Pág. 11**

8. **Bibliografía .... Pág. 11-13**

9. **Cuaderno de recogida de datos .... Pág. 13**

1. Antecedentes y justificación del proyecto

La diástasis de rectos abdominales (DRA) consiste en la separación del vientre de los dos rectos abdominales (distancia interrectos) a lo largo de la línea alba con división fibrosa y ensanchamiento de la misma (1,2). Las funciones de la línea alba son diversas, por un lado, mantener los rectos anteriores próximos, garantizar la estabilidad de la columna, pelvis y suelo pélvico e intervenir en la función de los músculos abdominales ya que sirve de anclaje anterior para los mismos (3 -5).

Durante el embarazo, La DRA ocurre de forma fisiológica debido a cambios elásticos hormonales en el tejido conectivo y a las tensiones mecánicas sobre la pared abdominal producidas por el crecimiento del feto y el desplazamiento de los órganos abdominales (6). Suele aparecer entre el segundo y tercer trimestre (6) y se resuelve de forma espontánea entre 1 y 8 semanas tras el parto (7,8). En algunas mujeres, no se produce esta resolución espontánea tras dar a luz, convirtiéndose entonces en una situación patológica que puede mantenerse años tras el parto (6,7).

En la literatura científica el ejercicio más estudiado para comprobar el comportamiento de la línea alba ha sido el crunch abdominal, encontrándose en la mayoría de los casos una disminución de la distancia interrectos (IRD) en comparación con el reposo (9 -13). En el caso de Mota et al. (12) y Chiarello et al. (9) demuestran que, en las mujeres con hijos, este ejercicio provoca disminución más acentuada de la IRD, que en las mujeres nulíparas. Con respecto a la activación de transverso, ejercicio recomendado para el tratamiento de la DRA (14,15), provoca un aumento de la IRD en la mayoría de los estudios (10,12,16), lo que podría deberse a la orientación de sus fibras que ayudarían a tensar y ensanchar la línea alba (1,12,17), favoreciendo un funcionamiento óptimo por la tensión creada (13).

La técnica abdominal hipopresiva fue descrita en 1980 por Marcel Caufriez, un fisioterapeuta belga (18). Según Caufriez los ejercicios hipopresivos, destinados inicialmente para mujeres en el periodo postparto, producen una caída de la presión abdominal a través de la aspiración diafragmática y esto a su vez provoca la contracción de los músculos del suelo pélvico y los músculos abdominales profundos (18,19).

La evidencia sobre el efecto de los hipopresivos es escasa, y la mayoría centrada en sus efectos sobre el suelo pélvico. Concluyendo varios autores que los ejercicios hipopresivos no aportan mayor beneficio que los ejercicios de suelo pélvico en cuanto a la función (20,21), aumento de la sección transversal (22), reducción del área hiato del elevador del ano (23) o en el prolapso de órganos pélvicos (24).

En cuanto al efecto de estos ejercicios sobre la pared abdominal todavía es más desconocido, pero Stüpp et al. (20) observaron la activación del transverso del abdomen durante la realización de un ejercicio hipopresivo medida mediante electromiografía de superficie. Esta activación del transverso del abdomen, tal y como se indica anteriormente podría tener efectos sobre la distancia interrectos

(10,12,16), por ello creemos que los ejercicios hipopresivos podrían tener también efecto sobre esta distancia.

Hasta el momento, según el conocimiento de los autores, tan solo existen dos ensayos clínicos aleatorizados que analizan el efecto de programas de ejercicios sobre la distancia interrectos en mujeres en el postparto. El primero de ellos se basa en el entrenamiento de la musculatura de suelo pélvico durante 16 semanas no encontrando efectos sobre la distancia interrectos (25). Más recientemente Thabet et al. (26) evaluaron el efecto de un programa de fortalecimiento de deep-core stability- durante 8 semanas hallando una disminución de la distancia interrectos tras el tratamiento.

2. Hipótesis del estudio

La realización de un programa de ejercicios hipopresivos tras el parto produce una mayor reducción de la distancia interrectos en comparación con la realización de un programa de ejercicios abdominales clásicos.

3. Objetivos

- Objetivo principal: Evaluar el efecto de dos programas de fortalecimiento abdominal (ejercicios abdominales clásicos *versus* ejercicios abdominales hipopresivos) sobre la distancia interrectos en mujeres en el postparto.

- Objetivos secundarios: Evaluar las posibles diferencias sobre el efecto de los programas en función de su historial obstétrico (edad materna, peso fetal, nº de partos, ganancia de peso en el embarazo)

4. Tipo de estudio

- Definición del diseño del estudio: Ensayo clínico de grupos paralelos y ciego.

5. Material y métodos

5.1 Ámbito de estudio: Salud y patología en el periodo postparto

5.2 Definición de los sujetos a estudio

Las participantes del estudio se seleccionarán de centros de atención primaria del área sanitaria de Vigo.

Selección y retirada de sujetos:

- Criterios de inclusión y exclusión. Como criterios de inclusión se establece tener una edad entre 25 y 40 años, haber dado a luz 8 semanas antes de comenzar la fase de tratamiento del estudio y aceptar participar en el mismo. Como criterios de exclusión, se eliminarán aquellas mujeres con hernia abdominal, embarazo y cirugías abdominales

- Criterios de retirada: las participantes podrán retirarse del estudio en cualquier momento de forma voluntaria. Además, será un criterio de retirada la ausencia a más de un 20% (2 sesiones) de las sesiones del programa de ejercicios que se llevará a cabo en cada uno de los grupos.

5.3 Captación y reclutamiento de los participantes

Las participantes del estudio se seleccionarán de los siguientes centros de atención primaria del área sanitaria de Vigo: Sárdoma, Matamá, Bembrive, Lavadores, Teis, Navia y Coia.

En las consultas previas al parto, la matrona de cada centro de salud explicará a las mujeres la finalidad de este estudio, se les invitará a participar si cumplen los criterios de inclusión que pueden ser evaluados antes de parto. Las pacientes que los cumplan deberán aceptar formar parte de este estudio firmando el consentimiento informado. Después del parto, se debe comprobar de nuevo si las mujeres cumplen los criterios de inclusión por ejemplo por el tipo de parto, que es algo que no se puede saber en las consultas durante el embarazo.

Las matronas de cada uno de los centros de salud encargadas de la recopilación de las participantes son:

- Maria Ameijeiras Canosa (CS Sárdoma, Matamá y Bembrive)
- Ana Isabel Costalago Herrera (CS Navia)
- Josefa Pereira Perez (CS Coia)
- Carmen López Videla (C.S Teis)
- Sara Arias Freire (CS. Coia)

5.4 Procedimiento de asignación/aleatorización

Se llevará una aleatorización simple a cada uno de los grupos. Una vez obtenido el consentimiento se aleatorizará a las pacientes a cada uno de los grupos según un listado de números aleatorios generado previamente

5.5 Justificación del tamaño muestral.

Se estableció un tamaño muestral de 10 participantes para cada grupo. La estimación del tamaño muestral se basó en el estudio de Sancho et al (10). en los que el tamaño del efecto estaba entre 0.43 y 0.25 de la distancia interrectos para las medidas supraumbilical e infraumbilical. Este tamaño muestral se calculó mediante el software G-Power 3.1 con un error alfa de 0,05 y una potencia de 0,95. Considerando un porcentaje de pérdidas de un 30%, finalmente se estimó un tamaño muestral de 14 participantes por grupo.

5.6 Variables principal y secundarias

Se utilizará como variable de efecto del tratamiento la diferencia de la distancia interrectos (DIR) antes y después de la intervención. El investigador que llevará a cabo las mediciones estará ciego no sabiendo a qué grupo pertenece cada una de las participantes. L as mediciones se llevarán a cabo en el centro de Salud de Navia, cuando exista disponibilidad de los espacios y previa autorización de la Gerencia del área de Salud de Vigo.

- Medición de la DIR: Las participantes se situarán en decúbito supino con las rodillas flexionadas a 90 º con los pies apoyados en la camilla y los brazos a lo largo del cuerpo. Después de recibir las instrucciones de realizar un crunch abdominal, se pide a los sujetos que separen la cabeza y eleven los hombros hasta que la escápula se despegue de la camilla.

Las imágenes de ultrasonido se registrarán con un escáner de ultrasonido (GE Logic-e; 4-12 MHz, transductor lineal de 39 mm; modo B), en una posición de reposo supino y una posición de activación muscular (contracción isométrica), 2 cm y 5 cm por encima del ombligo.

Para garantizar que las dos DIR medidas (2 cm y 5 cm por encima del ombligo) se realicen en la misma ubicación, se marcará la piel con un bolígrafo soluble en agua.

La recolección de imágenes de ultrasonido se realizará inmediatamente al final de la exhalación, según lo determinado por la inspección visual del abdomen siguiendo las recomendaciones de Teyhen et al. (27) Se prestará especial atención a la presión ejercida en la sonda de ultrasonido sobre el paciente para evitar la respuesta reflexiva de la musculatura abdominal (28).

Para la adquisición de imágenes por ultrasonido, el borde inferior del transductor será colocado sobre la marca realizada y se moverá lateralmente hasta que se puedan visualizar los bordes mediales de ambos músculos rectos anteriores. La orientación del transductor se ajustará para

optimizar la visualización de la imagen. Las imágenes de ultrasonido recopiladas se exportarán al formato DICOM para una medición posterior, analizadas por el mismo investigador, utilizando un código Matlab personalizado (Image Processing Toolbox, Mathworks Matlab, EE. UU.) Siguiendo los procedimientos descritos por Mota et al. (28). Existe una confiabilidad intra-evaluador buena en las imágenes de ultrasonidos de DIR, con valores de intervalo de confianza superiores a 0,90(27).

Se recogerán como variables independientes:

- Grupo al que se aleatoriza al paciente (grupo de ejercicios abdominales hipopresivos y grupos de ejercicios abdominales clásicos)

- Variables obstétricas (edad materna, peso fetal, nº de partos, ganancia de peso en el embarazo): estas variables serán administradas por las matronas a través de su historia clínica.

5.7 Descripción de la intervención

A partir de la semana 8 tras el parto se procederá a toma de medidas y a la intervención de los dos grupos. En primer lugar, se procederá a la toma de mediciones que se llevará a cabo en un día con el procedimiento explicado en el punto anterior. Después las participantes durante 6 semanas llevarán a cabo la intervención en cada uno de los grupos y posteriormente una vez pasen estas 6 semanas se llevará a cabo la toma de mediciones finales. Ninguno de los programas de fortalecimiento abdominal se ofrece como servicio habitual a las mujeres en el período postparto en los centros de atención primaria.

Descripción de la intervención

Grupo 1: Ejercicios de fortalecimiento abdominal clásico (Curl-up, sit-ups y leg-rise), ejercicios de equilibrio, movilidad general y ejercicios de fortalecimiento de miembros superiores.

Grupo 2: Ejercicios abdominales hipopresivos, ejercicios de equilibrio, movilidad general y ejercicios de fortalecimiento de miembros superiores.

En ambos grupos los programas de ejercicios tendrán una duración de 6 semanas, con una temporalización de 2 días por semana (1 hora por sesión), haciendo un total de 12 sesiones. Las intervenciones se llevarán a cabo en el centro de Salud de Navia, cuando exista disponibilidad de los espacios y previa autorización de la Gerencia del área de Salud de Vigo

5.8 Cronograma y fecha prevista de finalización. Distribución de las tareas entre los miembros del equipo investigador

La previsión es comenzar y terminar el estudio en el año 2022 siempre que las condiciones sanitarias lo permitan y se cuente con la aprobación del comité ético. La distribución de tareas de los investigadores es la siguiente:

- Iria Da Cuña Carrera: Desarrollo teórico del proyecto y organización del resto de investigadores. Aleatorización de las participantes a los grupos de tratamiento. Análisis estadístico de los datos y participación en la redacción del artículo de investigación derivado de la presente investigación.

- Eva Mª Lantarón Caeiro: Encargada de llevar a cabo el programa de ejercicios en el grupo de fortalecimiento abdominal clásico. Participación en la redacción del artículo derivado de la presente investigación.

- Mercedes Soto González: Encargada de llevar a cabo el programa de ejercicios en el grupo de ejercicios abdominales hipopresivos. Participación en la redacción del artículo derivado de la presente investigación

- Augusto Gil Pascoal: Participación en el desarrollo teórico del proyecto, en el análisis estadístico de los datos y en la redacción del artículo derivado de la presente investigación. Encargado de llevar a cabo las evaluaciones (pre y post intervención) a través de las mediciones ecográficas.

- Maria Ameijeiras Canosa: Captación de las participantes y recogida de las variables obstétricas.

- Ana Isabel Costalago Herrera: Captación de las participantes y recogida de las variables obstétricas.

- Josefa Pereira Perez: Captación de las participantes y recogida de las variables obstétricas.

- Carmen López Videla: Captación de las participantes y recogida de las variables obstétricas.

- Sara Arias Freire: Captación de las participantes y recogida de las variables obstétricas

5.9 Plan de análisis estadístico

La variable dependiente (DIR) se analizará utilizando pruebas estándar de normalidad (prueba de Shapiro-Wilk) para encontrar los criterios de normalidad (29). En el caso de una distribución normal de los datos, la diferencia en la DIR se analizará a través de un análisis de la varianza (ANOVA)

Para el análisis de las variables obstétricas y su relación con la DIR se utilizará el coeficiente de correlación de Pearson o Spearman.

El análisis principal se basará en la diferencia en la DIR pre- post intervención y la comparación entre grupos mediante un análisis de medidas repetidas. Se establecerá un nivel de significación de $p<0,05$ para todas las variables.

6. Aspectos ético-legales

6.1 Cumplimiento de Normas de Buena Práctica Clínica, Declaración de Helsinki, Convenio de Oviedo, así como normativa de protección de datos, manejo de historia clínica y otra normativa de aplicación según el tipo de estudio.

Los investigadores manifiestan su compromiso de adhesión a los convenios éticos y a las normas de buena práctica clínica, á la normativa actual de investigación y de protección de datos y a la confidencialidad de la información de las participantes del estudio, de acuerdo con la Ley 14/2007, Declaración de Helsinki y el Convenido de Oviedo y la Ley Orgánica 3/2018 de Protección de datos (LOPD e GDD)

6.2 Modelos de consentimiento informado.

Se aporta como documentación anexa a la solicitud el consentimiento informado y la hoja de información de la investigación.

6.3 Confidencialidad de la información recogida en el contexto del estudio.

La obtención, tratamiento, conservación, comunicación y cesión de sus datos se hará conforme a lo dispuesto en el Reglamento General de Protección de Datos (Reglamento UE 2016-679 del Parlamento europeo y del Consejo, de 27 de abril de 2016) y la normativa española sobre protección de datos de carácter personal vigente.

Los datos necesarias para llevar a cabo este estudio serán recogidos y conservados de modo seudonimizados (codificados). En este estudio solamente las personas del equipo investigador conocerán el código que permita saber su identidad.

Una vez que se finalice el estudio los datos serán conservados anonimizados, de forma que ni siquiera el equipo investigador podrá identificar a las participantes.

6.4 Póliza de seguro o justificación de su ausencia para los estudios experimentales

Las investigadoras que van a llevar a cabo la intervención poseen seguro de responsabilidad civil al encontrarse colegiadas por el Colegio Oficial de Fisioterapeutas de Galicia, lo cual es obligatorio a la hora de llevar a cabo una intervención con pacientes como se da en esta investigación.

6.5 Compromiso de publicación de los resultados

Los investigadores se comprometen a publicar los resultados obtenidos en el trabajo que se presenta.

7. Memoria económica y fuente de financiación

No existe ninguna fuente de financiación para el proyecto que se presenta.

8. Bibliografía

1. Axer H, Keyserlingk DG, Prescher A. Collagen fibers in linea alba and rectus sheaths. I. General scheme and morphological aspects. J Surg Res. 2001 Mar;96(1):127 -34.

2. Coldron Y, Stokes MJ, Newham DJ, Cook K. Postpartum characteristics of rectus abdominis on ultrasound imaging. Man Ther. 2008 May;13(2):112 -21.

3. Gilleard WL, Brown JM. Structure and function of the abdominal muscles in primigravid subjects during pregnancy and the immediate postbirth period. Phys Ther. 1996 Jul;76(7):750 -62.

4. Parker A, Millar L, Dugan S. Diastasis Rectus Abdominis and Lumbo-Pelvic Pain and Dysfunction-Are They Related? J Women´s Health Phys Ther. 2009;33(2):15 -22.

5. Lee DG, Lee LJ, McLaughlin L. Stability, continence and breathing: the role of fascia following pregnancy and delivery. J Bodyw Mov Ther. 2008 Oct;12(4):333 -48.

6. Boissonnault JS, Blaschak MJ. Incidence of diastasis recti abdominis during the childbearing year. Phys Ther. 1988 Jul;68(7):1082 -6.

7. Candido G, Lo T, Janssen P. Risk factor for diastasis of the recti abdominis. J Assoc Chart Physiother Women Health. 2005;97.
8. Keeler J, Albrecht M, Eberhardt L, Horn L, Donnelly C, Lowe D. Diastasis Recti Abdominis: A Survey of Women's Health Specialists for Current Physical Therapy Clinical Practice for Postpartum Women. J Women´s Health Phys Ther. 2012;36(3):131 -42.
9. Chiarello CM, McAuley JA, Hartigan EH. Immediate Effect of Active Abdominal Contraction on Inter-recti Distance. J Orthop Sports Phys Ther. 2016 Mar;46(3):177 -83.
10. Sancho MF, Pascoal AG, Mota P, Bø K. Abdominal exercises affect inter-rectus distance in postpartum women: a two-dimensional ultrasound study. Physiotherapy. 2015;101(3):286 -91.
11. Pascoal A, Dionisio S, Cordeiro F, Mota P. Inter-rectus distance in postpartum women can be reduced bay isometric contraction of the abdominal muscles: a preliminary case-control study. Physiotherapy. 2014;100:344 -8.
12. Mota P, Pascoal AG, Carita AI, Bø K. The Immediate Effects on Inter-rectus Distance of Abdominal Crunch and Drawing-in Exercises During Pregnancy and the Postpartum Period. J Orthop Sports Phys Ther. 2015 Oct;45(10):781 -8.
13. Lee D, Hodges PW. Behavior of the Linea Alba During a Curl-up Task in Diastasis Rectus Abdominis: An Observational Study. J Orthop Sports Phys Ther. 2016 Jul;46(7):580 -9.
14. Benjamin DR, van de Water ATM, Peiris CL. Effects of exercise on diastasis of the rectus abdominis muscle in the antenatal and postnatal periods: a systematic review. Physiotherapy. 2014 Mar;100(1):1 -8.
15. Liaw L-J, Hsu M-J, Liao C-F, Liu M-F, Hsu A-T. The relationships between inter-recti distance measured by ultrasound imaging and abdominal muscle function in postpartum women: a 6-month follow-up study. J Orthop Sports Phys Ther. 2011 Jun;41(6):435 -43.
16. Theodorsen NM, Strand LI, Bø K. Effect of pelvic floor and transversus abdominis muscle contraction on inter-rectus distance in postpartum women: a cross-sectional experimental study. Physiotheraoy. 2018 Oct;In press.
17. Grässel D, Prescher A, Fitzek S, Keyserlingk DGV, Axer H. Anisotropy of human linea alba: a biomechanical study. J Surg Res. 2005 Mar;124(1):118 -25.
18. Caufriez M. Gymnastique abdominale hypopressive. M.V. Editions. Bruselas; 1997.
19. Caufriez M. Rééducation Myostatique hypopressive. I:N:K. Bruselas; 1999.
20. Stüpp L, Resende APM, Petricelli CD, Nakamura MU, Alexandre SM, Zanetti MRD. Pelvic floor muscle and transversus abdominis activation in abdominal hypopressive technique through surface electromyography. Neurourol Urodyn. 2011 Nov;30(8):1518 -21.
21. Resende APM, Stüpp L, Bernardes BT, Oliveira E, Castro RA, Girão MJBC, et al. Can hypopressive exercises provide additional benefits to pelvic floor muscle training in women with pelvic organ prolapse? Neurourol Urodyn. 2012 Jan;31(1):121 -5.

22. Bernardes BT, Resende APM, Stüpp L, Oliveira E, Castro RA, Bella ZIKJ di, et al. Efficacy of pelvic floor muscle training and hypopressive exercises for treating pelvic organ prolapse in women: randomized controlled trial. Sao Paulo Med J Rev Paul Med. 2012;130(1):5 -9.

23. Resende APM, Torelli L, Zanetti MRD, Petricelli CD, Jármy-Di Bella ZIiK, Nakamura MU, et al. Can Abdominal Hypopressive Technique Change Levator Hiatus Area?: A 3-Dimensional Ultrasound Study. Ultrasound Q. 2016 Jun;32(2):175 -9.

24. Resende APM, Bernardes BT, Stüpp L, Oliveira E, Castro RA, Girão MJBC, et al. Pelvic floor muscle training is better than hypopressive exercises in pelvic organ prolapse treatment: An assessor-blinded randomized controlled trial. Neurourol Urodyn. 2018 Oct 12;

25. Gluppe SL, Hilde G, Tennfjord MK, Engh ME, Bø K. Effect of a Postpartum Training Program on the Prevalence of Diastasis Recti Abdominis in Postpartum Primiparous Women: A Randomized Controlled Trial. Phys Ther. 2018 Apr 1;98(4):260 -8.

26. Thabet A, Mansour A, Alshehri. Efficacy of deep core stability exercise program in postpartum women with diastasis recti abdominis: a randomised controlled trial. J Musculoskelet Neural Interact. 2018;

27. Teyhen DS, Gill NW, Whittaker JL, Henry SM, Hides JA, Hodges P. Rehabilitative ultrasound imaging of the abdominal muscles. J Orthop Sports Phys Ther. 2007 Aug;37(8):450 -66.

28. Mota P, Pascoal AG, Sancho F, Bø K. Test-retest and intrarater reliability of 2-dimensional ultrasound measurements of distance between rectus abdominis in women. J Orthop Sports Phys Ther. 2012 Nov;42(11):940 -6.

29. Portney LG, Watkins MP. Foundations of clinical research: applications to practice. 3rd ed. Upper Saddle River, NJ: Pearson/Prentice Hall; 2009.

9. Cuaderno de recogida de datos

La recogida de datos, se llevará a cabo en un documento Excel. La columna del grupo al que pertenecen las participantes será cubierta al final del estudio por la investigadora principal, ya que el investigador que lleva a cabo la evaluación estará cegado durante la toma de datos y cuando introduzca los datos en el documento Excel. Se anexa la hora de recogida de datos.
