# Supplementary material for: Effect of hypopressive and conventional abdominal exercises on postpartum diastasis recti: A randomized controlled trial
Source: PLoS One. 2024 Dec 12;19(12):e0314274. doi: 10.1371/journal.pone.0314274 (PMC11637234; doi:10.1371/journal.pone.0314274)
Supplement: S2 Protocol — (PDF) [file pone.0314274.s008.pdf]

Subscribe to DeepL Pro to translate larger documents.
Visit www.DeepL.com/pro for more information.

RESEARCH PROTOCOL

COMPARISON OF TWO ABDOMINAL EXERCISE PROGRAMS ON INTERRECTAL DISTANCE IN POSTPARTUM WOMEN

Iria Da Cuña Carrera

January 2022

- **Project title**: COMPARISON OF TWO ABDOMINAL EXERCISE PROGRAMS ON INTERRECTIVE DISTANCE IN POST-PARTUM WOMEN.

- **Protocol code**

- **Protocol version with date**

- **Promoter:** Iria Da Cuña Carrera

- **Principal Investigator:** Iria Da Cuña Carrera. Assistant Professor at the Faculty of Physiotherapy at the University of Vigo.

Contact information:
Iria Da Cuña Carrera.
E-mail: iriadc@uvigo.es. Phone: 986801758 /
652131637.
Faculty of Physiotherapy. Campus a Xunqueira s/n CP36005 Pontevedra

- **Collaborating researchers**
  - Eva M. Lantarón Caeiro (University of Vigo)
  - Mercedes Soto González (University of Vigo)
  - Augusto Gil Pascoal (Universidade de Lisboa)
  - Maria Ameijeiras Canosa (Sárdoma, Matamá and Bembrive Health Centers)
  - Ana Isabel Costalago Herrera (CS Navia)
  - Josefa Pereira Perez (CS Coia)
  - Carmen López Videla (C.S Teis)
  - Sara Arias Freire (CS Coia)

Index

1. **Background and justification of the project .... Page 4-5**
2. **Hypothesis of the study**...**Page 5**
3. **Objectives .... Page 5**
4. **Type of study .... Page 5**
5. **Material and methods .... Pages 5-9**
   5.1. Scope of study
   5.2. Definition of study subjects
   5.3 Recruitment of participants
   5.4 Allocation/randomization procedure
   5.5 Justification of sample size
   5.6 Primary and secondary variables
   5.7 Description of the intervention
   5.8 Timeline and expected completion date. Distribution of tasks among the members of the research team.
   5.9 Statistical analysis plan
6. **Ethical and legal aspects .... P. 10-11**
   6.1 Informed consent forms.
   6.2 Confidentiality of the information collected in the context of the study.
   6.3 Insurance policy or justification of absence for experimental studies
   6.4 Commitment to publish results
7. **Financial report and source of financing .... Page 11**
8. **Bibliography .... Pages 11-13**
9. **Data collection notebook .... Page 13**

1. Project background and justification

Diastasis of the rectus abdominis (DRA) consists of the separation of the belly of the two rectus abdominis (inter rectus distance) along the linea alba with fibrous division and widening of the same (1,2). The functions of the linea alba are diverse, on the one hand, to maintain the anterior rectus abdominis close together, to guarantee the stability of the spine, pelvis and pelvic floor and to intervene in the function of the abdominal muscles since it serves as an anterior anchorage for them (3 -5).
During pregnancy, ARD occurs physiologically due to hormonal elastic changes in the connective tissue and mechanical stresses on the abdominal wall produced by the growth of the fetus and the displacement of abdominal organs (6). It usually appears between the second and third trimester (6) and resolves spontaneously between 1 and 8 weeks after delivery (7,8). In some women, this spontaneous resolution does not occur after delivery, and it then becomes a pathologic condition that may persist for years after delivery (6,7).

In the scientific literature, the most studied exercise to check the behavior of the linea alba has been the abdominal crunch, finding in most cases a decrease in the inter-straight distance (IRD) compared to rest (9 -13). Mota et al. (12) and Chiarello et al. (9) show that, in women with children, this exercise causes a more accentuated decrease in IRD than in nulliparous women. Regarding transverse activation, an exercise recommended for the treatment of ARD (14,15), it causes an increase in DRI in most studies (10,12,16), which could be due to the orientation of its fibers that would help to tighten and widen the linea alba (1,12,17), favoring optimal functioning due to the tension created (13).

The abdominal hypopressive technique was described in 1980 by Marcel Caufriez, a Belgian physiotherapist (18). According to Caufriez, hypopressive exercises, initially intended for women in the postpartum period, produce a drop in abdominal pressure through diaphragmatic suction and this in turn causes contraction of the pelvic floor muscles and deep abdominal muscles (18,19).

The evidence on the effect of hypopressive exercises is scarce, and most of it is focused on their effects on the pelvic floor. Several authors conclude that hypopressive exercises do not provide greater benefit than pelvic floor exercises in terms of function (20,21), increase in cross-sectional area (22), reduction of the hiatus area of the levator ani (23) or pelvic organ prolapse (24).

As for the effect of these exercises on the abdominal wall is still more unknown, but Stüpp et al. (20) observed activation of the transversus abdominis during the performance of a hypopressive exercise measured by surface electromyography. This activation of the transversus abdominis, as indicated above, could have an effect on the interrectal distance.

(10,12,16), therefore we believe that hypopressive exercises could also have an effect on this distance.

So far, to the authors' knowledge, there are only two randomized clinical trials that analyze the effect of exercise programs on inter-straight distance in postpartum women. The first of these is based on pelvic floor muscle training for 16 weeks and found no effect on inter-straight distance (25). More recently, Thabet et al. (26) evaluated the effect of a deep-core stability-strengthening program for 8 weeks and found a decrease in interrectal distance after treatment.

2. Study hypothesis

Performing a program of hypopressive exercises after childbirth results in a greater reduction of the interrectal distance compared to performing a program of classic abdominal exercises.

3. Objectives

- Main objective: To evaluate the effect of two abdominal strengthening programs (classical abdominal exercises *versus* hypopressive abdominal exercises) on the inter-straight distance in postpartum women.

- Secondary objectives: To evaluate possible differences on the effect of programs depending on their obstetric history (maternal age, fetal weight, no. of deliveries, weight gain in pregnancy).

4. Type of study

- Definition of study design: Blinded, parallel group clinical trial.

5. Material and methods

5.1 Field of study: Health and pathology in the postpartum period.

5.2 Definition of study subjects

Study participants will be selected from primary care centers in the Vigo health area.

Selection and withdrawal of subjects:

- Inclusion and exclusion criteria. Inclusion criteria were to be between 25 and 40 years of age, to have given birth 8 weeks before beginning the treatment phase of the study and to agree to participate in the study. As exclusion criteria, women with abdominal hernia, pregnancy and abdominal surgeries will be eliminated.

- Withdrawal criteria: participants may voluntarily withdraw from the study at any time. In addition, absence from more than 20% (2 sessions) of the sessions of the exercise program to be carried out in each of the groups will be a criterion for withdrawal.

5.3 Recruitment of participants

The study participants will be selected from the following primary care centers in the Vigo health area: Sárdoma, Matamá, Bembrive, Lavadores, Teis, Navia and Coia.

In the pre-delivery consultations, the midwife at each health center will explain to the women the purpose of this study, and they will be invited to participate if they meet the inclusion criteria that can be evaluated before delivery. Patients who meet the criteria must agree to take part in this study by signing the informed consent form. After delivery, it should be checked again whether the women meet the inclusion criteria, for example by the type of delivery, which is something that cannot be known in the consultations during pregnancy.

The midwives from each of the health centers in charge of collecting the participants are:

- Maria Ameijeiras Canosa (CS Sárdoma, Matamá and Bembrive)
- Ana Isabel Costalago Herrera (CS Navia)
- Josefa Pereira Perez (CS Coia)
- Carmen López Videla (C.S Teis)
- Sara Arias Freire (CS. Coia)

5.4 Allocation/randomization procedure

A simple randomization will be performed for each of the groups. Once consent has been obtained, the patients will be randomized to each of the groups according to a list of random numbers previously generated.

5.5 Justification of sample size.

A sample size of 10 participants was established for each group. The estimation of the sample size was based on the study by Sancho et al (10), in which the effect size was between
0.43 and 0.25 of the inter-straight distance for the supraumbilical and infraumbilical measurements. This sample size was calculated using G-Power 3.1 software with an alpha error of 0.05 and a power of 0.95. Considering a loss rate of 30%, a sample size of 14 participants per group was finally estimated.

5.6 Primary and secondary variables

The difference in inter-straight distance (DIR) before and after the intervention will be used as the treatment effect variable. The researcher who will carry out the measurements will be blinded and will not know to which group each participant belongs. The measurements will be carried out at the Navia Health Center, when space is available and with prior authorization from the Management of the Vigo Health Area.

- Measurement of DIR: Participants will be positioned in the supine position with knees bent at 90° with feet resting on the table and arms alongside the body. After being instructed to perform an abdominal crunch, subjects are asked to separate the head and elevate the shoulders until the scapula detaches from the table.
Ultrasound images will be recorded with an ultrasound scanner (GE Logic-e; 4-12 MHz, 39 mm linear transducer; B-mode), in a supine resting position and a muscle activation position (isometric contraction), 2 cm and 5 cm above the umbilicus.

To ensure that the two DIR measurements (2 cm and 5 cm above the umbilicus) are taken at the same location, the skin is marked with a water-soluble pen.
Ultrasound image collection will be performed immediately at the end of exhalation, as determined by visual inspection of the abdomen following the recommendations of Teyhen et al. (27) Special attention will be paid to the pressure exerted on the ultrasound probe on the patient to avoid reflexive response of the abdominal musculature (28).
For ultrasound image acquisition, the lower edge of the transducer will be placed over the mark made and moved laterally until the medial edges of both anterior rectus muscles can be visualized. The orientation of the transducer will be adjusted to

optimize image visualization. The collected ultrasound images will be exported to DICOM format for further measurement, analyzed by the same investigator, using a customized Matlab code (Image Processing Toolbox, Mathworks Matlab, USA) following the procedures described by Mota et al. (28). There is a good intra-evaluator reliability in DIR ultrasound images, with confidence interval values higher than 0.90(27).

These will be collected as independent variables:

- Group to which the patient is randomized (hypopressive abdominal exercises group and classic abdominal exercises group).

- Obstetric variables (maternal age, fetal weight, number of deliveries, weight gain during pregnancy): these variables will be administered by the midwives through their clinical history.

5.7 Description of the intervention

From week 8 after delivery, measurements will be taken and the intervention of the two groups will be carried out. First, measurements will be taken, which will be carried out in one day with the procedure explained in the previous point. Afterwards, the participants will carry out the intervention in each of the groups for 6 weeks and then, once these 6 weeks have passed, the final measurements will be taken. None of the abdominal strengthening programs are offered as a regular service to women in the postpartum period in primary care centers.

Description of the intervention

Group 1: Classic abdominal strengthening exercises (curl-ups, sit-ups and leg-rise), balance exercises, general mobility and upper limb strengthening exercises.

Group 2: Hypopressive abdominal exercises, balance exercises, general mobility and upper limb strengthening exercises.

In both groups the exercise programs will have a duration of 6 weeks, with a timing of 2 days per week (1 hour per session), making a total of 12 sessions. The interventions will be carried out in the Navia Health Center, when there is availability of space and prior authorization of the Management of the Health Area of Vigo.

5.8 Timeline and expected completion date. Distribution of tasks among the members of the research team.

The study is expected to begin and end in 2022, provided that health conditions permit and the ethics committee approves. The distribution of tasks of the investigators is as follows:

- Iria Da Cuña Carrera: Theoretical development of the project and organization of the rest of the researchers. Randomization of the participants to the treatment groups. Statistical analysis of the data and participation in the writing of the research article derived from this research.

- Eva Mª Lantarón Caeiro: In charge of carrying out the exercise program in the classical abdominal strengthening group. Participation in the writing of the article derived from this research.

- Mercedes Soto González: In charge of carrying out the exercise program in the hypopressive abdominal exercises group. Participation in the writing of the article derived from this research.

- Augusto Gil Pascoal: Participation in the theoretical development of the project, in the statistical analysis of the data and in the writing of the article derived from this research. In charge of carrying out the evaluations (pre and post intervention) through ultrasound measurements.

- Maria Ameijeiras Canosa: Recruitment of participants and collection of obstetric variables.

- Ana Isabel Costalago Herrera: Recruitment of participants and collection of obstetric variables.

- Josefa Pereira Perez: Recruitment of participants and collection of obstetric variables.

- Carmen López Videla: Recruitment of participants and collection of obstetric variables.

- Sara Arias Freire: Recruitment of participants and collection of obstetrical variables

5.9 Statistical analysis plan

The dependent variable (DIR) will be analyzed using standard normality tests (Shapiro-Wilk test) to find the normality criteria (29). In the case of a normal distribution of the data, the difference in DIR will be analyzed through an analysis of variance (ANOVA).

The Pearson or Spearman correlation coefficient will be used for the analysis of obstetric variables and their relationship with the DIR.

The main analysis will be based on the difference in DIR pre-post intervention and the comparison between groups by means of a repeated measures analysis. A significance level of $p<0.05$ will be established for all variables.

6. Ethical and legal aspects

6.1 Compliance with Good Clinical Practice Guidelines, Declaration of Helsinki, Oviedo Convention, as well as data protection regulations, clinical history management and other applicable regulations according to the type of study.

The investigators declare their commitment to adhere to the ethical conventions and standards of good clinical practice, to the current research and data protection regulations and to the confidentiality of the information of the study participants, in accordance with Law 14/2007, Declaration of Helsinki and the Oviedo Convention and the Organic Law 3/2018 on Data Protection (LOPD and GDD).

6.2 Informed consent forms.

The informed consent form and the research information sheet are attached to the application form.

6.3 Confidentiality of the information collected in the context of the study.

The collection, processing, conservation, communication and transfer of your data will be done in accordance with the provisions of the General Data Protection Regulation (Regulation EU 2016-679 of the European Parliament and of the Council of 27 April 2016) and the Spanish regulations on the protection of personal data in force.

The data necessary to carry out this study will be collected and stored in a pseudonymized (coded) manner. In this study, only members of the research team will know the code that allows their identity to be known.

Once the study is completed, the data will be kept anonymized, so that not even the research team will be able to identify the participants.

6.4 Insurance policy or justification of absence for experimental studies

The researchers who are going to carry out the intervention have civil liability insurance as they are members of the Official College of Physiotherapists of Galicia, which is mandatory when carrying out an intervention with patients as in this research.

6.5 Commitment to publish results

The researchers undertake to publish the results obtained in the work presented.

7. Financial report and source of financing

There is no source of funding for the project being presented.

8. Bibliography

1. Axer H, Keyserlingk DG, Prescher A. Collagen fibers in linea alba and rectus sheaths. I. General scheme and morphological aspects. J Surg Res. 2001 Mar;96(1):127 -34.

2. Coldron Y, Stokes MJ, Newham DJ, Cook K. Postpartum characteristics of rectus abdominis on ultrasound imaging. Man Ther. 2008 May;13(2):112 -21.

3. Gilleard WL, Brown JM. Structure and function of the abdominal muscles in primigravid subjects during pregnancy and the immediate postbirth period. Phys Ther. 1996 Jul;76(7):750 -62.

4. Parker A, Millar L, Dugan S. Diastasis Rectus Abdominis and Lumbo-Pelvic Pain and Dysfunction-Are They Related? J Women's Health Phys Ther. 2009;33(2):15 -22.

5. Lee DG, Lee LJ, McLaughlin L. Stability, continence and breathing: the role of fascia following pregnancy and delivery. J Bodyw Mov Ther. 2008 Oct;12(4):333 -48.

6. Boissonnault JS, Blaschak MJ. Incidence of diastasis recti abdominis during the childbearing year. Phys Ther. 1988 Jul;68(7):1082 -6.

7. Candido G, Lo T, Janssen P. Risk factor for diastasis of the recti abdominis. J Assoc Chart Physiother Women Health. 2005;97.
8. Keeler J, Albrecht M, Eberhardt L, Horn L, Donnelly C, Lowe D. Diastasis Recti Abdominis: A Survey of Women's Health Specialists for Current Physical Therapy Clinical Practice for Postpartum Women. J Women's Health Phys Ther. 2012;36(3):131 -42.
9. Chiarello CM, McAuley JA, Hartigan EH. Immediate Effect of Active Abdominal Contraction on Inter-recti Distance. J Orthop Sports Phys Ther. 2016 Mar;46(3):177 -83.
10. Sancho MF, Pascoal AG, Mota P, Bø K. Abdominal exercises affect inter-rectus distance in postpartum women: a two-dimensional ultrasound study. Physiotherapy. 2015;101(3):286 -91.
11. Pascoal A, Dionisio S, Cordeiro F, Mota P. Inter-rectus distance in postpartum women can be reduced bay isometric contraction of the abdominal muscles: a preliminary case-control study. Physiotherapy. 2014;100:344 -8.
12. Mota P, Pascoal AG, Carita AI, Bø K. The Immediate Effects on Inter-rectus Distance of Abdominal Crunch and Drawing-in Exercises During Pregnancy and the Postpartum Period. J Orthop Sports Phys Ther. 2015 Oct;45(10):781 -8.
13. Lee D, Hodges PW. Behavior of the Linea Alba During a Curl-up Task in Diastasis Rectus Abdominis: An Observational Study. J Orthop Sports Phys Ther. 2016 Jul;46(7):580 -9.
14. Benjamin DR, van de Water ATM, Peiris CL. Effects of exercise on diastasis of the rectus abdominis muscle in the antenatal and postnatal periods: a systematic review. Physiotherapy. 2014 Mar;100(1):1 -8.
15. Liaw L-J, Hsu M-J, Liao C-F, Liu M-F, Hsu A-T. The relationships between inter-recti distance measured by ultrasound imaging and abdominal muscle function in postpartum women: a 6-month follow-up study. J Orthop Sports Phys Ther. 2011 Jun;41(6):435 -43.
16. Theodorsen NM, Strand LI, Bø K. Effect of pelvic floor and transversus abdominis muscle contraction on inter-rectus distance in postpartum women: a cross-sectional experimental study. Physiotheraoy. 2018 Oct;In press.
17. Grässel D, Prescher A, Fitzek S, Keyserlingk DGV, Axer H. Anisotropy of human linea alba: a biomechanical study. J Surg Res. 2005 Mar;124(1):118 -25.
18. Caufriez M. Gymnastique abdominale hypopressive. M.V. Editions. Brussels; 1997.
19. Caufriez M. Hypopressive myostatic rehabilitation. I:N:K. Brussels; 1999.
20. Stüpp L, Resende APM, Petricelli CD, Nakamura MU, Alexandre SM, Zanetti MRD. Pelvic floor muscle and transversus abdominis transversus activation in abdominal hypopressive technique through surface electromyography. Neurourol Urodyn. 2011 Nov;30(8):1518 -21.
21. Resende APM, Stüpp L, Bernardes BT, Oliveira E, Castro RA, Girão MJBC, et al. Can hypopressive exercises provide additional benefits to pelvic floor muscle training in women with pelvic organ prolapse? Neurourol Urodyn. 2012 Jan;31(1):121 -5.

22. Bernardes BT, Resende APM, Stüpp L, Oliveira E, Castro RA, Bella ZIKJ di, et al. Efficacy of pelvic floor muscle training and hypopressive exercises for treating pelvic organ prolapse in women: randomized controlled trial. Sao Paulo Med J Rev Paul Med. 2012;130(1):5 -9.
23. Resende APM, Torelli L, Zanetti MRD, Petricelli CD, Jármy-Di Bella ZIiK, Nakamura MU, et al. Can Abdominal Hypopressive Technique Change Levator Hiatus Area: A 3-Dimensional Ultrasound Study. Ultrasound Q. 2016 Jun;32(2):175 -9.
24. Resende APM, Bernardes BT, Stüpp L, Oliveira E, Castro RA, Girão MJBC, et al. Pelvic floor muscle training is better than hypopressive exercises in pelvic organ prolapse treatment: An assessor-blinded randomized controlled trial. Neurourol Urodyn. 2018 Oct 12;
25. Gluppe SL, Hilde G, Tennfjord MK, Engh ME, Bø K. Effect of a Postpartum Training Program on the Prevalence of Diastasis Recti Abdominis in Postpartum Primiparous Women: A Randomized Controlled Trial. Phys Ther. 2018 Apr 1;98(4):260 -8.
26. Thabet A, Mansour A, Alshehri. Efficacy of deep core stability exercise program in postpartum women with diastasis recti abdominis: a randomised controlled trial. J Musculoskelet Neural Interact. 2018;
27. Teyhen DS, Gill NW, Whittaker JL, Henry SM, Hides JA, Hodges P. Rehabilitative ultrasound imaging of the abdominal muscles. J Orthop Sports Phys Ther. 2007 Aug;37(8):450 -66.
28. Mota P, Pascoal AG, Sancho F, Bø K. Test-retest and intrarater reliability of 2-dimensional ultrasound measurements of distance between rectus abdominis in women. J Orthop Sports Phys Ther. 2012 Nov;42(11):940 -6.
29. Portney LG, Watkins MP. Foundations of clinical research: applications to practice. 3rd ed. Upper Saddle River, NJ: Pearson/Prentice Hall; 2009.

9. Data collection notebook

Data collection will be carried out in an Excel document. The column of the group to which the participants belong will be covered at the end of the study by the principal investigator, since the researcher conducting the evaluation will be blinded during data collection and when entering the data in the Excel document. The time of data collection is attached.
